# Medical Student Professionalism Narratives: A Thematic Analysis and Interdisciplinary Comparative Investigation

**DOI:** 10.1186/1471-227X-11-11

**Published:** 2011-08-12

**Authors:** Aaron W Bernard, Matthew Malone, Nicholas E Kman, Jeffrey M Caterino, Sorabh Khandelwal

**Affiliations:** 1 The Department of Emergency Medicine, The Ohio State University College of Medicine, Columbus OH, USA

## Abstract

**Background:**

Professionalism development is influenced by the informal and hidden curriculum. The primary objective of this study was to better understand this experiential learning in the setting of the Emergency Department (ED). Secondarily, the study aimed to explore differences in the informal curriculum between Emergency Medicine (EM) and Internal Medicine (IM) clerkships.

**Methods:**

A thematic analysis was conducted on 377 professionalism narratives from medical students completing a required EM clerkship from July 2008 through May 2010. The narratives were analyzed using established thematic categories from prior research as well as basic descriptive characteristics. Chi-square analysis was used to compare the frequency of thematic categories to prior research in IM. Finally, emerging themes not fully appreciated in the established thematic categories were created using grounded theory.

**Results:**

Observations involving interactions between attending physician and patient were most abundant. The narratives were coded as positive 198 times, negative 128 times, and hybrid 37 times. The two most abundant narrative themes involved *manifesting respect *(36.9%) and *spending time *(23.7%). Both of these themes were statistically more likely to be noted by students on EM clerkships compared to IM clerkships. Finally, one new theme regarding *cynicism *emerged during analysis.

**Conclusions:**

This analysis describes an informal curriculum that is diverse in themes. Student narratives suggest their clinical experiences to be influential on professionalism development. Medical students focus on different aspects of professionalism depending on clerkship specialty.

## Background

A growing body of literature supports the notion that professionalism is largely learned in a latent, implicit, and experiential manner [1,2]. Classroom didactics, skills workshops and other explicit activities of the formal curriculum of medical school take a back seat to what has been termed the *hidden *and *informal *curriculum [1,2]. The *hidden *curriculum is defined as the organizational structure and culture that influences learning. This includes the customs, norms, and rituals of day-to-day activities such as rounding. The *informal *curriculum is the interpersonal experiences between students and teachers, other students, and patients. Learning through observations of and interactions with roles models is part of the *informal *curriculum [1,2].

A thorough understanding of these day-to-day influences is important for advances in professionalism education to occur. Recently, a thematic analysis of professionalism narratives from students on an Internal Medicine (IM) clerkship helped unveil these experiences [3]. We aim to pick up where this study left off in a new setting; the Emergency Department (ED). Our primary goal is to further the understanding of the latent curriculums through an analysis of professionalism narratives written during an Emergency Medicine (EM) clerkship. More specifically, we aim to explore these narratives in order to gain an understanding of what aspects of professionalism students choose to reflect upon while rotating in the ED.

Secondarily, we aim to explore differences in the informal curriculum between EM and IM clerkships. The Association of American Medical Colleges recommends the utilization of various clinical settings in undergraduate medical education. This is felt to promote the development of the core clinical skill competencies; one of which is professionalism [4]. It is currently unclear if all aspects of professionalism are equally learned across the spectrum of clinical settings or if certain aspects are uniquely learned in specific environments. To the best of our knowledge, no prior work has attempted to compare student experiences regarding professionalism between clinical settings.

## Methods

### Study Design

This was a retrospective analysis of medical student professionalism narratives. The study was reviewed by The Office of Responsible Research Practices at The Ohio State University (OSU) and was deemed exempt from Institutional Review Board review.

### Study Setting and Population

The study population was fourth year medical students at one medical school completing a mandatory, four week, clerkship in EM between July 2008 and April 2010. The clerkship consists of a centralized didactic experience and a de-centralized clinical experience. Students complete sixteen, eight hour, shifts at one of thirteen different hospitals. All hospitals are within sixty miles of the college of medicine but vary substantially in a variety of characteristics; patient demographic (age, race, socio-economic status), ED census volume, location (rural, suburban, urban), staffing models, and educational mission (number and type of residencies, if any).

As part of the centralized didactic and independent learning requirements, students are given a professionalism assignment. Students are instructed to observe and record observations demonstrating professional or unprofessional behavior in the ED, while working clinically, that resulted in a better understanding of professionalism. There is no emphasis on either positive or negative events. Each student was required to post at least one narrative on an online discussion board during their EM clerkship. In addition, each student was required to post at least one response comment regarding another student's narrative in order to encourage conversation. The discussion board was accessible via password access to rotating students and only to the posts of that month. Narratives were not screened or edited and were immediately available to be read upon posting. The discussion board, while private and confidential, was not anonymous in that posts were identifiable by author. No attending physicians had access to this except the course director who did not view the posts until the grades for that month were complete and finalized.

### Study Protocol and Data Analysis

Narratives were de-identified by an administrator not associated with the investigation prior to the beginning of data analysis. The analysis of narratives was conducted primarily using established thematic categories from prior research [3]. These thematic categories were not adjusted as stipulated a priori to allow for statistical comparisons between investigations. Researchers read the narratives in an iterative manner and determined where they belonged in the established thematic categories. Narratives were simultaneously analyzed using standard grounded theory to determine if additional themes emerged not fully appreciated by the established thematic categories [5,6]. These new themes were noted and recorded separately.

Two investigators (AB and MM) independently reviewed the narratives. Multiple readings of each narrative were performed to gain a thorough understanding of the content and appropriate placement of narratives within the established thematic categories. If a new understanding of either the narratives or the established thematic categories was achieved, all narratives were re-read to ensure proper placement.

After a full review of all narratives, the two investigators conducted a collaborated review of each narrative. In cases where disagreement of coding existed the investigators would stop and discuss the coding in detail. The key language that led to the categorical decision was discussed and the narratives were further reviewed to achieve a consensus coding. In the event that a consensus could not be reached due to disagreements between investigators, third and fourth investigators (NK and SK) were used to mitigate. Further group analysis with all four investigators was used to determine a final coding of these disputed narratives. It has been noted in previous research that a single narrative may contain multiple themes [3]. To resolve this issue, any narrative containing multiple themes would have each theme categorized separately in order to prevent loss of data.

The narratives were further independently analyzed in various ways. First, the narratives were categorized as primarily positive, negative or as a "hybrid". Several types of hybrid posts were observed. This included narratives describing two events that contrasted and also narratives where the student presents the situation as professionally ambiguous, without a "right" or "wrong" way to handle it. A final type of hybrid included a "damage and repair" narrative [3]. In these, the participant initially acted in an unprofessional manner but then acted professionally by correcting the situation. It should also be explained that in a few rare instances, a story contained multiple thematic categories where one category was considered "positive" and the other "negative." In this situation, the narrative was not categorized as a "hybrid." Instead, these narratives were coded separately as a positive in one category and a negative in the other.

The second additional categorization of narratives was by individuals involved in the interaction (such as doctor/patient, doctor/student, nurse/patient, etc.). Individuals were only counted if they were directly involved in the observed event or were critical to the event. For instance, if a staff member made a comment directed at a patient behind the patient's back then both the staff member and the patient were counted, although the patient was not physically present.

Finally, a quantitative analysis was done to compare our results to those by Karnieli-Miller et al. regarding an IM clerkship [3,7]. All analyses for this section were completed by one investigator [JC] using STATA v11 (STATACorp, College Station, TX). For the quantitiative analysis, proportions with 95% confidence intervals were calculated for narrative types (positive, negative, or hybrid), persons involved in the narratives, overall theme domain (medical-clinical vs. teaching-learning), and for the 14 individual theme types. We used chi-square analysis to make comparisons between our results and those of Karnieli-Miller. *P*-values < 0.05 were considered significant. For all chi-square analyses involving a table larger than 2 × 2 and where a significant difference was detected, we calculated adjusted standardized residuals (ASR) to determine which cells made significant contributions to the rejection of the null hypothesis [8]. Cells with adjusted standardized residuals whose absolute value was greater than 1.96 were considered to be significant contributors as this corresponds to *p *< 0.05.

## Results

The results are presented in three sections for clarity. First the descriptive data is presented. The second section is the thematic analysis of the posts. This includes the frequency that narratives were coded into the established thematic categories as well as a description of one new theme that emerged during narrative review. The second section also includes direct representative quotes to better appreciate the findings of the investigation. More extensive examples of narratives can be found in Additional File 1. Finally, the comparative data is presented in section three.

### Descriptive data

The data collected includes 377 narratives from the 404 fourth year medical students rotating at 13 different central Ohio emergency departments recorded from July 2008 through April 2010. Approximately 10% of the narratives demonstrated two major themes, which resulted in a total count of 413 thematic elements coded for the 377 narratives.

The most frequent participants in the narratives were attending physicians, who appeared in 276 narratives (73.2%). The other individuals involved in the narratives were patients (184 posts; 48.8%) family members (58; 15.4%), residents (25; 6.6%), nurses (28; 7.4%), consultants (15; 4.0%), "the team" (26; 6.9%), other physicians (8; 2.1%), other students (1; 0.3%), prehospital personnel (paramedics, etc.) (6; 1.5%) and all other individuals combined (e.g., physical therapists, laboratory technicians) (12; 3.2%) and interns (2; 0.5%).

Of the 377 narratives posts, 198 were coded as positive, 128 were coded as negative, and 37 were coded as hybrid. 12 narratives were general comments without a specific story and 2 were coded separately as both positive and negative but in two different thematic categories.

### Thematic Analysis

The established categories used for thematic analysis involved two major domains. The first was the medical-clinical interactions domain, which included observations of faculty and staff interactions with patients, families, coworkers, and colleagues. The second domain focused on the teaching and learning environment, which included the students' experiences as learners in the clinical setting [3]. The analysis revealed that 383 thematic elements (92.7%) were categorized under the medical-clinical interactions domain, while 30 thematic elements (7.3%) fell under the teaching and learning environment domain. Table 1 presents the major themes, sub-themes, and positive, negative, and hybrid stories. Table 2 presents the same data for the teaching and learning environment domain.

**Table 1:** Thematic Content of Professionalism Narratives Within the Medical- Clinical Interaction Domain

Theme	%	Subcategory	#Pos	#Neg	#Hyb	#Tot*
1. Manifesting respect or disrespect in clinical interactions with patients, families, colleagues, and coworkers	33.7	a. Respecting patients'/families' decisions, wishes, or needs	14	8	9	31
		b. Acting respectfully with patients/families in challenging situations	12	7	3	22
		c. Having disrespect toward/from colleagues	1	14	2	17
		d. Treating patient as a person and not a disease carrier	1	1	0	2
		e. Using appropriate language/interaction with a patient/colleague	1	10	1	12
		f. Being respectful to stigmatized populations	4	9	2	15
		g. Using inappropriate humor/comments (behind the patient's back)	0	21	1	22
		h. Criticizing others	2	1	0	3
		i. Showing disrespect toward the profession/negative attitudes	0	3	0	3

2. Managing communication challenges with patients and families	16.7	a. Handling difficult situations/conversations with patients/families	25	3	6	34
		b. Communicating in a caring and compassionate way	4	3	0	7
		c. Communicating with angry/resistant patients or families	22	0	0	22

3. Demonstrating responsibility, pride, knowledge, and thoroughness	9.8	a. Displaying responsibility, honesty, and integrity	3	12	0	15
		b. Acquiring updated knowledge/lifelong learning	0	0	0	0
		c. Thoroughly investigating patients' problems	8	7	1	16
		d. Striving toward excellence	6	0	0	6
		e. Acknowledging your limitations	0	0	0	0
		f. Having pride in work	0	0	0	0

4. Spending time taking care of patients, patients' education, and understanding	26.0	a. Spending time to talk and answer patients'/families' needs for information and support	54	8	3	65
		b. Spending time with patients, listening respectfully, learning their history and concerns	17	3	2	22
		c. Communicating in a level/language that patients can understand	2	0	0	2
		d. Taking full responsibility for patient care and informing health care providers and caregivers	7	2	0	9

5. Going above and beyond, caring, and altruism	6.6	No subcategories	20	3	2	25

6. Communicating and working in teams	5.6	No subcategories	9	12	0	21

7. Unclear stories	3.2	General comments without a specific story				12

*The total number of narratives exceeds 377 because a single story may have been classified more than once under different thematic categories.

**Table 2:** Thematic Content of Professionalism Narratives Within the teaching and learning environment domain

Theme	%377	Subcategory	#Pos	#Nag	#Hyb	#Tot*
8. Creating an (un)welcoming environment	4.0	a. Respecting colleagues/learners from lower hierarchies	2	9	1	12
		b. Being tolerant to mistakes, providing constructive feedback and evaluations	1	0	1	2
		c. Included and acknowledged as a medical student	0	0	1	1
		d. Judgmental environment	0	0	0	0

9. Capitalizing on teaching opportunities	3.4	a. A leader who teaches--asks questions, explains, spends time, learns	6	1	1	8
		b. Using opportunities to teach values and manners	2	0	0	3
		c. Giving safe and structured responsibilities	0	0	2	2

10. Learning from peers	0.0	a. Fellow student teaching and helping other students (demonstrating teamwork)	0	0	0	0
		b. Fellow student relating to a patient as a person	0	0	0	0
		c. Taking care of fellow colleagues	0	0	0	0

11. Dealing with attending/staff or self expectations	0.3	a. Unclear expectations from attending and staff	0	1	0	1
		b. Self expectations as a professional	0	0	0	0

12. Paying attention to students' needs	0.0	a. Attuned to students' personal needs/life situation	0	0	0	0
		b. Caring for students	0	0	0	0

13. Having space to conduct private conversations	0.3	No subcategories	0	1	0	1

14. Demonstrating honesty and integrity	0.0	No subcategories				0

*The total number of narratives exceeds 377 because a single story may have been classified more than once under different thematic categories.

The most common theme noted in narrative analysis was *manifesting respect or disrespect in clinical interactions with patients, families, colleagues, and coworkers*. Often times the content of the narratives that fell under this theme was focused on the appropriate use of the ED. As one student explains:

I have been impressed during each one of my shifts how respectful the attendings remain when faced with patients who have made poor choices or who are presenting to the ED when they really should be going to a family physician or staying home. Although they might be annoyed, the attendings patiently work up the patient, explain the disease process, and discharge the patient home with clear instructions.

This narrative demonstrates the complexity of the narratives and the breadth of professionalism described. While the narrative demonstrates many aspects of professional behavior from being thorough, to responsibility, to spending time giving explanations, the main focus of the student is the respect given to patients who chose to use the ED for primary care. Repeatedly, students describe similar situations where parents bring young children in for mild fever or where adult patients express the need for refills on chronic care medication, all while physicians and staff respect their decisions and clinical wishes.

The second most common theme noted in narrative analysis was *spending time taking care of patients, patients' education, and understanding *was common. One student noted the following;

We had a 11 year old girl whose mom brought her in because she had been running low grade fevers, coughing, and just feeling overall under the weather for a couple weeks. We started to explain to the mom that this was a virus, her daughter was otherwise fine, the fevers were not dangerous, and it is safe to go home, but the mom broke in and wanted a complete explanation of why this is not appendicitis. My attending could have gotten short with this mother, but instead he sat down and patiently explained our physical exam, what we look for, and why we ask the questions we did. He took a full 20 minutes allaying this mom's fears and convincing her that we really did rule out all the things she was afraid her daughter might have. If he had not taken the time to explain everything, the mom would have likely been back the next day because her concerns had not been addressed.

Again, like the previously discussed narrative, this story involves multiple aspects of professionalism from respect to communication. The key feature however that the student was trying to portray here was the time spent answering questions and giving the mother adequate support.

Narratives within different themes focused on various content. One content area that was prevalent in EM was pain management. In this content area students would often comment about the appropriate use of narcotic medication and interactions with patients with drug seeking behavior. The narratives of this content were categorized in a variety of themes. Some were positive narratives that could be classified under the theme of *spending time *and others were negative narratives, that were classified under *using inappropriate comments (behind a patients back)*. Perhaps the most common theme noted for this content focus was *managing communication challenges with patients and families*. In one narrative, a student describes the professional way one physician approaches difficult conversations about pain and prescription narcotic use:

I had an attending who felt very strongly about not giving out any pain medications to people who were clearly in the ED just to get narcotics. We had one case where the person had visited the ED 10 times in the past year and over half of those times she was discharged with narcotics. He (the attending) used the Ohio substance abuse monitoring site and found that the patient receives 120 pills/month from a family doctor and appears to supplement through the ED 3-4 times per month. He politely went in and explained to her that she has a family doctor who prescribes narcotic medicine for her and he was uncomfortable doing so. He explained it was not his job to refill narcotic medications when the patient has a physician who prescribes them and that she should make an appointment for the next day. The patient was surprisingly understanding.

This narrative is similar to the previously discussed respect story, with a patient using the ED in a way the student feels is inappropriate. However, this narrative focuses more heavily on the aspects of how the conversation took place and the communication techniques used to professionally convey his message.

Professionalism narratives were infrequently categorized under the teaching and learning environment domain. Within this domain, two themes were notable. The first, *creating an (un)welcoming environment*, contains predominantly negative stories. One student writes:

It was my first shift in the ED, which my attending had already grumbled about when she first (met) me, and it happened to be a really busy day. Unfortunately I had been waiting to staff a patient for a while, and she was the only attending around so I approached her. She was standing, working on a computer, so I walked up and stood several feet away, waiting for a break in what she was doing. I didn't say anything because I didn't want to interrupt so I just waited. She eventually turned to me and says, "You need to chill out!" and angrily turns back to her computer. I understand she was busy but I think there could have been a nicer way to tell me I needed to staff my patient with the other attending.

The other theme within the teaching and learning environment domain worth noting is *capitalizing on teaching opportunities*, which had a majority of positive narratives. Another student explains:

I was very impressed by the dedication to excellence shown by one attending. Although most attendings will offer brief pearls in order to redirect residents or students when they are staffing patients, I rarely see attendings go beyond this level. During one shift I was surprised when one doc called together all the residents and myself for teaching. He thought that this point was important enough that it warranted breaking the hectic pace of the ED.

These two narratives offer completely different attending behaviors when teaching is involved. In the first instance, the environment created by the attending behavior was disruptive to the student while in contrast the second offered an example of an attending enthusiastic about education and the importance of training the next generation of physicians.

Narrative analysis revealed one new theme that was not appreciated in the established thematic categories. Student narratives often described incidents of involving *cynicism*. One student writes:

By the end of my first shift, the cynicism and skepticism that I was hearing from the staff in the ER was starting to rub off on me. This continued on my next 3-4 shifts. It was on my 5^th ^shift that I believe it went too far. The attending went to interview the patient but a minimal history could be taken as their was an obvious language barrier and the patient was having trouble answering questions with the pain he was in. When we left the room the attending told me that, "he doesn't have anything wrong with him. These people always come in for little aches and pains." We did a little testing on this gentleman. No imaging. Whether the attending had seen this 100 times before with no pathology involved, this could be the one time the patient had mesenteric ischemia for example. While I believe it's okay to have a little level of cynicism and skepticism in the ER, you should not let it interfere with your level of care.

In this narrative, the student was obviously upset at the about the type of care this patient received due to issues of cynicism and skepticism. Further, this story demonstrates the importance of narration from a student's perspective. In this situation, the physician may have felt it more appropriate to dedicate his time to higher risk patients but this was not at all what the student perceived. Throughout the narratives the importance of the student's perspective on the narratives become evident. Students repeatedly describe situations they find inappropriate or unprofessional whereas experienced physicians may disagree.

### Comparative Data Analysis

In examining the relative proportions of narrative types present (positive, negative, hybrid), chi-2 analysis revealed no significant difference between our data and the data from Karnieli-Miller's work (*p *= 0.081) [3,7]. In examining persons cited in the narratives, we identified a greater number of persons cited per narrative (1.7 people cited per narrative versus 0.6 people cited per narrative). The overall chi-square analysis revealed a significant difference (*p *< 0.001) in the type of persons cited with the difference attributed to attendings (ASR = 6.82), residents (ASR = 7.06), consultants (ASR = 2.32), and other students (ASR = 2.71). The EM students in our study were more likely to reference attendings then the IM students in Karnielli-Miller, et al (42.0% vs. 13.7%). EM students were less likely to reference residents (3.7% vs. 19.0%), consultants (2.4% vs. 6.0%), and other students (0.2% vs. 1.8%).

When examining overall theme domains, EM students were significantly more likely to cite the medical-clinical domain (92.7%, 95% CI 89.8-95.0%) than IM students (82.3%, 95% CI 77.6-86.4%)(*p *< 0.001). When examining the 14 specific thematic categories the overall *p *value for the chi-square was < 0.001. Examination of ASRs revealed significant contributions from the following three thematic categories: *manifesting respect *(ASR = 2.01), *spending time *(ASR = 3.10), and *learning from peers *(ASR = 3.71). A fourth category, *demonstrating responsibility*, was near significance (ASR = 1.91). EM students were more likely to cite *manifesting respect *(30.8% vs. 23.9%) and *spending time *(23.7% vs. 14.4%) than IM students. EM students were less likely to cite *demonstrating responsibility *(9.0% vs. 13.4%) and *learning from peers *(0.0% vs. 3.3%).

## Discussion

This analysis describes an informal curriculum that is diverse in themes. Student narratives are vivid, detailed, and suggest their clinical experiences to be influential on professionalism development. This is consistent with prior research [3,9,10].

The specific aim of the study was to better understand the aspects of professionalism that students choose to reflect upon while completing an EM clerkship and how that differs from students on an IM clerkship. It appears students focused on attending behavior more frequently and resident behavior less frequently while on the EM clerkship. This may simply be related to exposure as many of the EM clerkship sites did not have residents present. However, this finding is important in that it highlights the need for variety in clinical settings during undergraduate medical education [4].

The domain of medical-clinical interaction was more frequently reflected upon then the teaching and learning domain for both EM and IM clerkships. However, EM clerkship students had an even greater affinity to reflect upon the medical-clinical interaction domain. It is unclear why this is the case. The ED is a relatively unique clinical setting. The patient population is heterogeneous, their problems are acute and undifferentiated, and the number of new patient encounters is high. The work environment is somewhat chaotic and unpredictable and patient care is provided in a multi-disciplinary, team-based manner [11,12]. Perhaps this unique setting and its contrast to the IM clerkship setting accounts for the differences noted in narrative domains.

Differences of frequency of specific themes within each domain was also noted. Statistical analysis suggested narratives of *manifesting respect *and *spending time *to be more prominent on EM clerkships [3,7]. The prominence of the *spending time *theme in EM narratives is particularly interesting. These narratives were overwhelming positive. Perhaps students did not expect this behavior in the clinical setting of a fast paced ED. Thus, when they experienced this unexpected behavior it was noticed and deemed worthy of reflection. It is difficult to know with certainty why reflective focus seemed to vary between EM and IM clerkships. However, the difference alone is notable and further highlights the need for multiple clinical environments in undergraduate medical education [4].

Understanding of the informal curriculum and differences that exist between clerkships may help educators engage students and optimize learning [13]. Reflective exercises have been demonstrated to improve knowledge acquisition and clinical skills [14-16]. To encourage diversity of reflection, prevent redundant exercises, and to maximize the use of experiences by clinical settings, educators may want to consider giving greater focus and direction to reflective exercises.

During the thematic analysis of ED narratives one new theme emerged regarding cynicism. A prior analysis of professionalism narratives specific to the ED also found issues of cynicism to be prominent in the ED setting [17]. Medical students and other professionals have noted that a major problem with their education is a failure of role models to live up to the standards set forth by the college of medicine [18]. This investigation highlights that problem again. Narratives of physicians appropriately interacting with "drug seeking" patients were very common, but so too were lapses in professionalism. Together with the problematic theme of cynicism this work suggests areas of potential improvement for Emergency Physicians. Prior work has been done at the institutional level to address global issues of professionalism with mixed results [19,20]. Promoting institutional changes to the professionalism culture needs new approaches [21]. Perhaps, targeting specific issues based on practice setting can make these programs more effective. We hope Emergency Physicians use the data presented here to make appropriate changes to achieve optimal professionalism in the ED.

## Limitations

The major limitation of this work was the comparison of two specialties not at the same institution. There were also subtle differences in instructions given to students regarding the writing of narratives [3]. Finally, our work focused on narratives from fourth-year medical students while the comparative data was primarily from third-year medical students [3]. Prior reports describing changes in student empathy and views of professionalism between years highlight this limitation [22,23]. A further study limitation is the inherent difficulty of performing scientific investigations regarding the topic of professionalism. This stems from a lack of clear and precise definitions of what exactly professionalism is in clinical practice [24].

## Conclusions

This analysis describes an informal curriculum that is diverse in themes. Student narratives suggest their clinical experiences to be influential on professionalism development. Medical students focus on different aspects of professionalism depending on clerkship specialty.

## Competing interests

The authors declare that they have no competing interests.

## Authors' contributions

AWB designed the study, performed the qualitative analysis, and drafted the manuscript. MM designed the study, performed the qualitative analysis, and drafted the manuscript. NK performed the qualitative analysis and revised the manuscript. JC performed the quantitative analysis and revised the manuscript. SK designed the study, performed the qualitative analysis, and revised the manuscript. All authors read and approved the final manuscript.

## Pre-publication history

The pre-publication history for this paper can be accessed here:

http://www.biomedcentral.com/1471-227X/11/11/prepub

## Supplementary Material

Additional file 1 **Selected Direct Representative Narratives**. Representative narratives for each of the thematic categories from the 2008-2009 academic year are presented. Click here for file
