# Supplementary material for: Medical Student Professionalism Narratives: A Thematic Analysis and Interdisciplinary Comparative Investigation
Source: BMC Emerg Med. 2011 Aug 12;11:11. doi: 10.1186/1471-227X-11-11 (PMC3166891; doi:10.1186/1471-227X-11-11)
Supplement: Additional file 1 — Selected Direct Representative Narratives. Representative narratives for each of the thematic categories from the 2008-2009 academic year are presented. [file 1471-227X-11-11-S1.DOC]

**Additional File 1: Select Direct Representative Narratives**

The narratives below are from the first year of study 2008-2009. They are copied as they are found in the blog with a title followed by a narrative. The only significant editing was to remove any identifiable variables regarding patients, hospitals, or staff. Some spelling was corrected.

**Professionalism Narratives: Medical – Clinical Interaction Domain**

**Main Theme: Manifesting respect or disrespect in clinical interactions with patients, families, colleagues, and coworkers**

**Sub-category: Respecting Patients’/families decisions, wishes, or needs**

Leaving AMA : About a week ago I saw a middle-aged, diabetic patient coming in with acute SOB.  When I asked the patient what brought her in, she said she was having an asthma attack and that she also had some tightness in the middle of her chest.  After assuring myself that the patient was not in immediate danger, I asked her a few more questions and then presented to the attending.  We thought that in addition to some breathing treatments, this patient warranted a cardiac evaluation (EKG, troponin) and admission for a stress test given her age and history of DM.  She was given pain control as well as a small dose of benzo because she said she had a history of anxiety.  Around the same time, one of the nurses provided us with a print-out of a discharge summary from one of this patient's previous ED visits.  This discharge summary made it obvious that patient had been seeking drugs in the past.  We also reviewed her medical records and saw no mention of asthma. After the labs, x-ray, and breathing treatment had been done, the attending returned to tell the patient about the plan for admission to rule out anything cardiac.  The patient refused this admission, making us wonder if her presentation was another way of seeking pain and/or sedative medication.  The attending very clearly spelled out in a nonjudgmental way that leaving was her choice but against his medical advice. Overall, I feel the attending handled this situation very professionally by both recommending a thorough work-up in a patient with risk factors but a history of drug-seeking and clearly explaining the process of leaving AMA.

Angry Patients: At XXXX ER one day there was a very angry mom who was upset because she'd been waiting for two hours with her 3mos old son. Her son had been hospitalized and intubated at XXX hospital for 10 days just a month ago with bronchiolitis and she was really scared that he was having trouble breathing again. She was very angry and told the attending and I that she felt like her son wasn't important to us since it had taken us so long to get back to see her. She said that she hated the hospital and if she had any other options she would take her son somewhere else. Meanwhile, her son was definitely having some breathing trouble and we gave him two breathing treatments. He calmed down somewhat after the second treatment but was still retracting and wheezing. My attending said that with most patients in this situation, he'd like to give him a couple more treatments and maybe watch him overnight in obs given his history. But, since the mom was so upset and the kid didn't look too bad and mom assured us that she would take the baby to the PCP in the morning, we let them go home. At the time it seemed to make sense to me the way things played out, but in retrospect I wonder if the best interests of the patient weren't perhaps somewhat compromised by the fact that his mom was so upset. Should her anger have changed the management of this patient?

Demanding Patients: I recently encountered this scenario.  A 55 year old woman comes to the ER with excruciating right back pain, sharp in nature, radiating towards the flanks.  She was sure something was wrong, maybe her 'kidneys.'  Her urinalysis was normal and she was afebrile.  On exam, the pain was not reproducible with palpation.  However, my attending still felt it was musculoskeletal in nature, and did not feel it was worrisome.  However, the patient and her husband demanded a CT scan.  The attending professionally discussed the pros and cons of getting a CT scan, and ultimately agreed to order the scan because of the family was adamant that the scan was necessary. In this case, the patient turned out to have a herniated disk, the cause of her pain.  However, there are many cases where patients are not satisfied with the diagnosis given (ie a patient saying that an anxiety attack couldn't be the cause of her chest pain because she doesn't have any stress) or demand more tests be done.  When do we go on our gut feeling (based on clinical judgment) about a diagnosis and when do we give in to patient's demands for unnecessary procedures and/or medications etc (assuming it is not a life-threatening situation)?  And when do we trust patients to know when something just isn't right?  We should also considering the increasing attention given to radiation exposure with multiple unnecessary tests and risk of cancer.

Privacy and Professionalism: At XXX Hospital ED this week, I was able to independently suture a laceration for the first time.  I was nervous, but a suture tech was there with me and helped keep the 7 yo boy calm during the procedure.  Just as I was getting started and the boy was calming down, a nurse came in the door and asked me about a couple pending orders for another child.  She wanted to know when they would be un-pended so she could get started on them.  I told her that the attending knew of the orders, and she was the one who had to cosign them so they would be active.  Then the nurse mentioned the other child's first name and asked me what the plan of care was going to be.  I felt distracted because I was suturing, and the child in the room was getting agitated.  I also felt it was inappropriate to talk about another patient's care while I was doing a procedure.  Not wanting to say those things to the nurse, I briefly told her the plan of care. Since the patient the nurse was asking about was not in urgent need, I wonder if it would have been better to ask me about the orders after the suturing was completed.  I also think it would have been better to only refer to the other child by room number rather than first name, if the case had to be discussed.  Looking back, I don't think I made the right decision to discuss the plan of care, and should have asked her if we could talk about it in a few minutes when I was out of the room.  Could I and the nurse have handled this better?

Addiction and Autonomy: A 23 year old female patient presented with severe 10/10 right flank pain, tearfulness, dysuria and CVA tenderness. She was diagnosed in the ED with pyelonephritis was prescribed antibiotics. The attending physician then asked the patient if she would like anything for the pain, which was clearly causing her distress. She replied that she wasn't sure if she should take anything, as she had a history of substance abuse. She stated that her abuse began with alcohol, marijuana, and pain medications and later included meth, crack, and IV heroin use. She also stated that she had been "clean" for over five years, to which her older sister, present in the room, agreed. The attending then told the patient that he would give her whatever she was comfortable with, including any narcotics. She said that she probably needed something, but was admittedly nervous about taking any addictive medications. At this point, the attending suggested "a few Vicodin and Motrin," to which the patient agreed.  This situation evoked an interesting ethical situation. The patient had real pain, and narcotics were probably indicated. However, given this patient's history and forthright apprehension, is it OK to grant the patient full autonomy in regards to her pain control? Was this the best treatment for the patient's long-term health?

Patients right to refuse care: I recently saw a patient in the ED who was complaining of severe abdominal pain.  It was noted in her history that she had been in the ED two weeks earlier and treated for suspected PID.  During that first visit she had refused a CT of the abdomen.  After examining the patient during this visit (including a pelvic) it was hypothesized that she most likely had failed treatment and developed a tuboovarian abscess.  A transvaginal ultrasound did not show either ovary so again a CT was ordered. The patient became extremely angry because of the amount of time she had spent in the ED that day and demanded to go home without waiting for the CT to be done. The new attending on shift went in to examine the patient and decided that she did not have an acute surgical abdomen and decided that since she had received an IV cephalosporin while in the ED that she could be sent home with a script for oral doxy. I found this to be an interesting situation because the patient had very recently failed treatment with the same antibiotics and it was explained that the CT would likely give us a definitive diagnosis, for which surgical drainage would be the best option, and the attending still had to respect the patient's decision to leave and allow her to go without the full workup.

Another DNR Story: XXX's story reminded me of a DNR issue I saw unfold in the ER. We had a female patient with mental status changes brought in by squad from a nursing home. The nursing home thought that she may have had a massive stroke. On arrival, the patient had a rightward fixed gaze and was minimally responsive to pain or other stimuli. This was a definite deterioration from her baseline. In her nursing home paperwork was a DNR-CC signed by the health care power of attorney. The attending was confused as to why a patient who was DNR-CC was brought to the ED at all. She called the nursing home and the best answer she could get was that sending the patient to the ED was what the family wanted. The attending called the health care POA, who turned out to be the patient's daughter. The attending explained the patient's symptoms to the daughter and then explained that we had paperwork signed by her saying that we were not to do anything except keep her comfortable. The daughter, for whom English was not a first language, was distraught. She explained she did not know about any such paperwork, that she had merely signed what someone had put in front of her. She asked that the order be reversed and that we do all we could to help her mother. I was appalled that the daughter had been asked to sign a document she did not fully understand. Part of me questions if the nursing home knew that the daughter was sort of unaware as to what she was signing; hence, the sending of the patient to the ER in the first place. Maybe I'm a bit cynical though.

Special Treatment: This month I am at XXX’s ED, and on three occasions, I have observed special treatment given to children whose parents are physicians. In the first situation, one of the ED attendings received a referral phone call for a patient whose father was one of the pediatric surgery attendings. Upon immediate arrival of the patient at the ED, this attending assigned himself to see the patient and was on his way to the patient's room as soon as she was wheeled in. In this way, he bypassed the usual system of having the patient be seen by the ED residents first. In the second situation, I overheard one of the residents remark to another resident that a particular inbound patient was the child of one of XXX head surgeons, so he was sure this would be an "attending only" ED visit. In the third situation, I myself had signed up to see a patient, a 7-year-old female with wheezing and cough/cold symptoms, who was a level 4 acuity according to XXX's system (1 is most acute, 5 is least acute). I had already written down the basic information and was just about to see this patient when I was called over by one of the attending physicians, who promptly informed me that he was "un-assigning me from the patient" because she was the child of one of XXX's ICU attendings. He then went to see the patient himself. Is this special treatment of children of physicians valid and professional, or does it go against values of social justice and equal treatment of all patients? Have others experienced similar situations, and why does this special treatment occur? I'd appreciate any thoughts on this matter...

Preconcieved Ideas: Last week a female came in with complaints of increased menstrual cramping and bleeding since the placement of Essure devices two years ago. The pain had been getting worse with each cycle and was now unbearable. She was bleeding profusely and was understandably concerned. Before my attending went to see her, he told me that there would be nothing wrong with the patient and conveyed his annoyance that he even had to see her. After I presented her case, he did concede to ordering a pelvic U/S which demonstrated that the Essure devices had migrated from her fallopian tubes to her endometrial cavity, the obvious source of her extreme pain. After some time had passed, the patient requested a second dose of pain medication as the first dose had worn off. When I informed my attending, he stated that he would not give her any more pain medication because it was taking too much energy from him. I was saddened by his lack of concern and compassion for the patient. I believe that his preconceived ideas about the patient influenced how it approached her.

Is Everyone Really Drug Seeking?: Throughout this rotation I have met many patients with many different types of pain.....mild, severe, perceived, real, pretend....the list can go on. One attending I have worked with is convinced that everyone who walks through the door is drug seeking and will deny him/her adequate relief until medical proof of the pain is given. This proof often comes at the cost of having a patient wait a long time in pain for the results to come back. I know we have to deny narcotics to those who are malingering, but we also need to relieve those who are truly suffering. Since pain is so subjective, where do you draw the line between great acting and true pain?

Morals Schmorals: I am currently working at XXX and I never really thought about possible morality differences at the institutions I am working in.  However because XXX is a Catholic hospital it seems like some of the doctors have different treatment options when it comes to caring for patients.  I have run into this the most involving OB/GYN care of patients.  This was especially true involving one patient.   She was a 30 y/o female with a history of menometrorrhagia for 1 year.  She came in because of her intense pain as well as her bleeding.  She said that though she had an OB/GYN at XXX because he was catholic "he did not want to take away her ability to procreate by doing an ablation or D and C.  He did prescribe her birth control pills though.  It seems like this has happened several times when certain doctors’ judgments on whether to offer an elective D and C for abortion or prescribe contraceptives to young women is based on their beliefs.  I was wondering what people think our role is in these situations.  Should we treat the patient as they wish to be treated even though it goes against our moral code or should we refuse?

End of Life: Just today there was a patient over 90, with multiple medical conditions, who came in septic and in cardiac arrest.  The patient was revived from PEA and made stable.  Her family wanted full code and said this is what the patient wants.  I did not hear about a living will.  However, the patient has a medical POA who was ademant that her mother be full code.  I could see the attending did not agree with the POAs decision; however, he did not impose his will on her.  He gave a great effort to both caring for the patient and to informing the family of her current medical situation.  This is a hard situation to navigate as it is easy to impose one's own will onto the patient and her family.  And, it is very possible the family is not acting in the best interest of the patient.  Moreover, a patient is using up limited medical dollars, physician time, and facilities who has a short duration and impaired quality of life.  Nevertheless, I feel the doctor did the right thing by honoring the POA's wishes and providing the best, most unbiased care possible

HIPPA: While passing the elevators in the hospital, I heard several doctors nearby discussing a patient's test results. I didn't stop to listen to the conversation, so I don't know whether or not they used names, but considering the combined hours we have spent doing HIPPA quizzes each year, (from which we have learned that elevators, cafeterias, hallways, etc., are off-limits when it comes to discussing patient information), it would have been nice to see the professionals that we work under being a bit more careful. I know that doctors are probably used to being in a constant state of discussion when it comes to patients, and this was probably just an oversight, but I believe that the effort should always be made to protect patient privacy to the fullest extent possible. No one ever said that professionalism would be easy all of the time. It does require an effort.

Testicular Fortitude: The XXX ER can be very busy on a weekend evening between 3 and 11 P.M.  On one such night, a 40 year-old gentleman came in to the ED with testicular pain of several hours duration.  He was clearly nervous about the nature of his complaint, about telling his medical history (which did involve STDs and recent unprotected intercourse), about his homosexual lifestyle, and the entire interaction was exceedingly awkward.  I was understanding in speaking to this patient, reassuring, my H&P blazoning with well-cultivated Patient Centered Medicine skills.  The attending physician listened to my presentation, saw the patient again with me, and we began a simple workup for the usual suspects.  This is all backstory and ultimately irrelevant, but it's interesting. The patient seems nice enough, merely uneasy. Attending is gone.  As I leave the patient's "room," he tells me he is thankful that the physician seeing him is not another physician in the Emergency Department, with whom he seems to have had some kind of unpleasant interaction during a previous ER visit.  So strong are his feelings, indeed, that the physician in question is a "butcher, and I would not having f-ing seen him, and this goddamn hospital is ..." and so on.  Fair enough.

When Do We Say No?: One issue that I keep running into and that i am sure most of you have experience, is that of the drug seeker patient.  For the past 2 weeks, we have had several repeat offenders coming with various complaints only to satisfy their drug habit.  One of my ER attending showed me an article of how much 9 individuals cost a texas hospital over a month time and believe it or not it ranged in the millions of our tax payers dollars... Sure, some our novice drug seekers complaints are sometimes easy to dismiss without very much testing, however the more savvy ones do cost us a lot: in time, money and yes in our faith in people... So when do we say no? Where is the limit to our hippocrates oath? And how do we implement protocols to stop this abuse of the system? And how do we continue to do no harm?

Treating Acute Pain in those with Chronic Pain: Recently a wheelchair bound female with chronic pain issues (2/2 extensive surgeries related to a genetic disorder), under the care of a PPM doc, came into the ER after falling out of her wheelchair onto the sidewalk, complaining of excruciating hip and sacrum pain.  The pain was so severe that it inhibited her from laying flat to be able to obtain imaging to assess for any fractures. According to the patient, she underwent a cholecystectomy the week prior that required her to increase usage of her pain meds--a circumstance that her PPM doc had not anticipated-- and the surgeon was uncomfortable adding to her already high doses of meds so recommended she see her PPM doc, who was unable to get her in any sooner (her appt was 4 days away from this ER visit).  The ER doc refused to give her any more pain meds because she could not document any injury.  In fact, she acknowledged privately to me that the 2mg IM dilaudid she gave the patient would be ineffective due to her high tolerance.  The patient left the ER in pain and feeling like she was not taken seriously. Chronic pain issues are a challenge to all physicians and some may agree that the ER doc was appropriate in not sending a Rx for pain meds home with the patient but knowingly under treating the patient while she was in the ER was completely inappropriate.

**Sub-category: Acting respectfully with patients/families in challenging situations**

Attitudes Toward Agitated Patients: I recently witnessed the following scenario during my medic ride along.  22 yo AAF following a failed suicide attempt at a local university.  She had mass texted her friends that she was going to commit suicide, and was found in her car by university police with her left wrist slashed with a small razor blade and a half-full bottle of gin in the passenger seat. She was intoxicated, combative, and violently resistant.  It took four large men to get her out of her car into the ambulance.  The general sentiment was that she had done this to get attention.  As a result, however, the medic on the team neglected to obtain appropriate vitals and stated on the intercom as he rolled his eyes that her blood pressure was "120/80".  Hospital security was on hand in the ED to help transfer her to a hospital bed and to immediately secure her in four point restraints.  There was one guard that was quite forceful, and literally jumped and sat on top of her to pin her down.  After she was secured, he deliberately stepped hard on her thigh as he got off.  Haldol was administered at a much later time. There were multiple lapses in professionalism during this encounter.  My question to you guys:- What incentives can be used to ensure EMS personnel report reliable information?
- Who advocates for the patient in the prehospital/EMS hand-off setting?

Lapse in Professionalism: I was working the other day and saw a patient presenting with back pain.  As I was presenting my case to the attending, one of the nurses was sitting nearby and noted some inconsistencies in the story I was conveying and what the patient had told her initially.  The attending went to speak with the patient, and came back with more inconsistencies.  The attending then had me go into the room with them, told the patient that they were starting to get ‘really frustrated’ and needed to clear up the inconsistencies.  The patient was requestioned and again gave a different response than what I had initially been told.  The attending rolled their eyes at the patient, turned to me and said “Is that what they told you?”  I felt extremely uncomfortable and didn’t think it was necessary to act in such a confrontational manner.  I replied that “it’s not what they had said initially, but maybe it’s just due to some miscommunication”.  Throughout the rest of the day the attending was pleasant and respectful to all of the other patients that came in, including the particular patient involved in this situation.  It was just a momentary lapse of professionalism, but there was no need to create a possible strain on the patient-doctor relationship by creating an ‘us versus them’ atmosphere between the medical team and the patient.

Handling Questionable Complaints: The other evening, we had a patient come into the ED at XXX Hospital with a primary complaint of "menstrual cramps".  As this patient was 22 years old, we figured that this was nothing new to her, and were questioning why she was coming into the ED.  (As an aside, her grandfather, who had stage IV cancer, came in at the same time for pleuritic chest pain.  We figured she would not have come in if it weren't for the fact that her grandfather was coming in anyway).  Before I went to talk to her, I discussed the complaint with the attending and how it should be handled.  He said, "I don't know anything about this patient.  She doesn't have a history of coming into the ED, so I don't know if this visit is her somehow trying to get narcotics.  Go and talk to her and get the story.  Before she gets any chance to ask anything about pain medications, bring up that studies have shown NSAIDs to be more effective against menstrual pains than narcotics." When I did talk to her, it was pretty much what we suspected--she was having cramps, but wouldn't have done anything about it if it weren't for the fact that her grandfather was coming into the ED anyway.  She had never taken anything for cramps before, and was grateful for the 800 mg motrin we gave her. I think the attending handled this well.  He didn't automatically label her as a drug-seeker who needed to be dealt with aggressively and removed from the ED, but instead treated her as a responsible adult who didn't know much about proper treatment for her condition and would benefit most from education.

2 Year Old Presenting Full Code: Today a 2 yo pt with Russell-Silver Syndrome arrived via EMS to XXXs ED in full code status.  She has asystole in 2 leads and was nonresponsive to attempts of chest compressions and multiple doses of Epinephrine.  I thought the staff at XXX's was very professional in handling the code as they allowed the patient's mother to come into the room to spend some time with her child after she passed and they provided adequate social work and pastoral care services to the patient's family.

Withholding Care: While my overall experience in the ED so far has been positive, I thought I would share with everyone a situation I observed where I questioned the professionalism of the medical team. A few days ago we had a patient brought in by the squad after he fell and broke his leg.  When EMS arrived the patient was very agitated and combative, as he had been drinking pretty heavily.  When we went into the room, the patient started yelling profanities at us and insulting us.  Eventually the patient calmed down and we were able to assess his injury.  During the assessment the patient was in a lot of pain.  When we left the room, the nurse asked the attending physician what we were going to give him for pain and the attending responded that we weren't going to give him anything and that he was going to "let him suffer for a while" because he was being such a jerk. While I understand that my attending was upset by the patient's rude behavior, I don't think it was the right decision to withhold pain meds from a patient who had a legitimate injury and was in real pain.  I was wondering if anyone else has experienced anything like this at their sites?

I’ll Choose the Blue Pill: Despite the unfortunately common "lapses in judgment" I have been witness too the past two weeks, I chose to address a scenario (a shift to be exact) demonstrating truly exemplary behavior.   During my overnight shift this weekend at XXX, we had the pleasure of interacting with some upstanding (read belligerent) patients admitted with intoxication (eg. alcohol, cocaine, "some blue pill," and "powder this dude dumped out of a bag which burned like crazy").  These patients provided highly compelling cases demonstrating why Doctoring in the 21st century may truly be for the cynic at heart.  Despite the obvious skepticism of the medical staff, the attending I was working beneath treated both patients with the utmost respect and without ridicule despite the obvious flaws in judgment each had delivered and the nurses' tangible desire to verbally chastise both.  I was truly impressed with his professionalism and his behavior is worthy of commendation (however I still will not mention his name).

Bumping Uglies: Recently a nurse at the ED was checking on a patient being worked up for abdominal pain. This patient had a fairly extensive workup so there was cause to believe his pain was real rather than imagined. Yet when she attempted to enter his room the door had been propped nearly shut with a chair. Through the partially open door the patient was observed to attempt to cover himself and his girlfriend jumped back and did the same. The nurse displayed exemplary judgment by not bringing up the rather painfully obvious situation and merely asked if the patient required anything. She then left the patient when he verbalized no response and documented her visit in a fashion similar to the above. There was no judgment, no condemnation and no attempt at negative implication in her documentation, which I found to be highly professional of her, especially given the situation. The patient later left the ED for a smoke with his girlfriend's assistance. I do not know if he ever returned, though my suspicion is that he did not.

The only lapse of professionalism here is that the gossip machine spread the incident around the ED like lightning. While the incident is certainly cause for concern as to the severity of the patient's complaint (and hilariously funny) it shouldn't become a generally known incident for anyone without direct contact with the patient for a medical reason, especially when the patient is still in the ED. Yes, the patient demonstrated extreme lack of judgment, but again it is not appropriate to spread gossip while a patient is being cared for and easily identifiable. Once the patient has been cared for and is no longer in the ED it may be appropriate to use the incident as a teaching point with no identifying information.

Positive Example: I've been really warmed to see how ER docs treat many of the patients who arrive due to overdose. We've had a few patients come into the ER this past week having taken overdoses of medications, and it's great to see how compassionate all of the physicians are in their treatment. There hasn't been any condescending language before or after the interview, which is a change from previous rotations. It's been great to see the staff take the time to talk to patients and help them find the right services without judgment or negativity.

Your money or your Life: Last week, I came across a situation that I thought put the patient in a rather awkward position.  While the attending and I started getting a history from a patient with acute chest pain radiating to her back, we were interrupted by the person who obtains insurance/financial information. She was nice enough, but it was a pretty strange situation to watch this person obtain the patient's financial info as the attending and I watched the patient suffering in acute chest pain.  Now, I understand that hospitals don't run on hopes and dreams alone, and that this patient's financial info needed to be obtained eventually.  Having said that, though, I think that this scenario sets up a very strange power dynamic, where the patient may have perceived us as demanding money before treatment--neither legal nor ethical in an emergency setting.  And certainly not professional. The attending commented that he has worked at XXX for many years and had never encountered that situation.  So, perhaps it was nothing more than an isolated occurrence.  Nonetheless, it was a fairly curious, thought-provoking event.

Letting Irritation Get the Better of You: I'm at a rural hospital, and like anywhere else, we get our share of med seekers. Most times we know who they are (OARRS, past ED visits) and that guides some of our decision making (offer NSAID, but refuse narcotic, etc). I saw one doc getting more and more annoyed with a patient who was complaining of some sort of pain (low back, abdominal, i'm not sure), and kept demanding IV pain medication. She had a definite history of med seeking, but despite that, the doc went ahead and ordered a dose of IV narcotics (I think to get her to shut up and leave). The nurses were unable to get an IV, despite multiple attempts. The patient continued to demand the IV med, and it seemed every 5 minutes, a nurse would come to the doctor and say, "Patient X is asking for meds." The doctor became very exasperated, and said something to the effect of, "Fine, if she wants IV meds, I'll put an EJ in her, we'll see if she likes that!" He proceeded to go to the patient, and without any explanation of what he was going to do, he put the bed into trendelenburg, palpated her EJ, and abruptly placed an IV there. The patient was crying and frightened at this point. I know she was a med seeker, and I know that the exam/tests ruled out any emergent issue, and could not find reason for her pain, and it was annoying to be constantly asked for pain meds, but I thought there must have been a better way to deal with this.

**Sub-category: Having disrespect toward/from colleagues**

Pressure Cooker: I worked with an attending last week that was a very competent physician who possessed all the qualities of a true professional.  Towards the tail end of his shift however, the ER pretty much blew up with patients and we were all struggling to keep our heads above water.  My attending managed to keep his composure while caring for the flash flood of patients that was spilling through the ER doors but finally lost it while trying to dictate cases from earlier in the day.  Every time he started a dictation something new would require his attention.  The last straw for him was when a nurse brought him an EKG to be signed off on just as he was restarting a dictation for what seemed like the 6th or 7th time.  He slammed the phone down on the workstation desk that he was at, snatched the EKG from the nurse without saying a word, read it and then angrily signed off on it.  I can't honestly say that I wouldn't have behaved the same way if put in his shoes but I think it's definitely helpful for us all to take a couple minutes to breath when we feel like we're about to lose it.  Although this attending displayed some unprofessional behavior, he did the professional thing later on by apologizing to the nurse after he had cooled down a bit.  Just remember to breath everyone.

Resident Poor Example: Today I witnessed a poor example of professionalism. There was some confusion about the discharge of a patient, a patient in room 18. His status on the track board was changed to "discharge" when it shouldn't have been. The nurse politely asked the resident about it. The resident snapped her head off, yelling at the nurse about the discharge status, when infact, it was the resident who must have changed it to discharge. I'm assuming She didn't want to take the blame. Some time passed, and the status still had not changed. So the nurse again politely asked the resident if it was okay to discharge room 18. This time the resident said, in an unpleasant tone, "room 18? I don't know anything about room 18. That's not my patient. You asked me about room 17 earlier!." In fact, the nurse did not ask about room 17 earlier, as I was a witness. The resident continued to blame the nurse for the confusion and stormed off in disgust.  I found the resident's actions very unnecessary and just plain rude. I say, treat the nurses with respect!

Bogus Consult: I'd heard it while on specialty services, "why am I consulted for this? Don't they realize this is non op?", or, "this doesn't have to be seen in the middle of the night, it can wait until morning" but now in the midst of our ED immersion experience, I realize there are two sometimes conflicting sides to this.    A drunk patient with a freshly fractured R mandible came into the ED overnight, took off his hoody, loitered outside his room in the hall, all the while unsavory.  There was emphasis therefore to get him seen, treated, and moved out.  Plastic surgery was covering facial trauma, and the resident was called in from home.  The resident had difficulty examining the patient for the stated reasons, and was not able to effectively dispo the patient, but handled it well. What I question was when the Plastic surgery day team came to the ED to follow up, one of the day residents cavalierly addressed the ED attending, paraphrased "If the patient is uncooperative and we can't get a good exam, you should think about this before you call in our resident from home".  Obviously everyone likes to sleep; alternately, most residents claim they also like to field consults made in earnest.  I now realize from this experience that the ED has its own priorities to treat patients expeditiously and maintain a safe environment.  I also consider that from the ED physician perspective, perhaps specialists have focused exam techniques and insight which allows them to assess even noncooperative patients effectively. My feeling is the day team resident could have considered the ED's perspective and helped to dispo this patient when possible, instead of complaining and viewing him as the ED's problem to deal with until willing to cooperate.

Rude Consultant: I made a call to a consultant and when I began to speak with them, was extremely short with me and blurted out questions in a condescending manner, then again abruptly cut me off saying "Put the attending on the phone".  This person proceeded to be rude with the fellow as well.  I believe this demonstrated a lapse in professionalism because of the manner in which the person addressed us may have affected the further treatment of the patient, who happened to be a complex case that required some more expertise in that area, and they acted as if "Why are you bothering me with this issue?"

Treatment of PA’s and CNP’s: I was at XXX and as part of the process of admitting the patient the PCP or specialist of the patient was often notified about the admission plan and asked if there was anything special they would like ordered or who we should admit to if there was no preference listed in the system. However, as part of their role the PA would often make these calls for patients they had seen and staffed with the attending. Some physicians and practice groups refused to talk with anyone but a physician though. I thought this was somewhat unprofessional to so disparage fellow professionals that you refuse to even listen to them tell you about a patient you worked up and instead force a busy attending to try and get all the pertinent data for what often amounted to a phone call of less than 60 seconds. I wondered if anyone else had experience working with physician extenders and how they were treated by other physicians in the system.

Consult Woes: One of the things I've found least enjoyable about learning the practice of medicine is how poorly consults are typically handled.  I'm sure by now we've all been on a service where we've dealt with "stupid" consults, where it seems that the consulter is just trying to dump his or her work on some poor consultee.  But the other day I was introduced to a different problem with consults that I found quite frustrating. A middle-aged woman had been seen at my site several days before my shift for a dislocated elbow and associated olecranon fracture.  On this day, she came back with complaints of pain and swelling in her elbow and forearm that had us concerned for a compartment syndrome.  This resulted in a consult to the orthopaedic hand team, and an attending and senior resident from the service came down to the ER to evaluate the patient.  They did a thorough exam, Dopplered pulses, and determined confidently that there was no impending compartment syndrome.  They then asked if I could place a splint on the patient and send her home.  All of this was fine and good up to this point ... it was an appropriate consult that was handled well by all parties involved.  However, here's where things got silly.  The ED attending I was working with--despite being the one who'd placed the consult to the "experts" in the first place--decided that the ortho guys were wrong.  As a result, she decided to get an arterial duplex study, check a D-dimer to rule out a clot (both of which came out negative), and then discharged the patient without even placing her in a splint like they'd wanted. I found this to be very frustrating for a couple of reasons.  First, why even place the consult if you're not going to respect the consultee's opinion?  I of course fully support "thinking for yourself" (a consultant can obviously be wrong), but this attending seemed to dismiss their opinion right out of hand.  Second--and even more troubling--when the data shook out that my attending was indeed the one who was wrong, she didn't even follow ortho's management requests!  It was as if she couldn't admit to being incorrect, and so had to do things "her way" regardless of what the data showed.  Anyone else see this sort of stubborn behavior before?

Professionalism Amongst Physicians: I recently observed an ER resident attempting to consult another service for their input and evaluation of a certain patient.  The ER resident gave a thorough presentation that described the problem and clearly stated what kind of examinations/test that he was requesting of the consulting service.  The resident from the consulting service proceeded to tell him how she had been to ED the previous day to see a patient and that she didn't think her service was needed then, and questioned why she was being consulted for this new patient.  She then informed the ER resident that she would get then in an hour because she didn't think it was urgent, and was also very rude/condescending in her demeanor.  I think we must remember that we are team, no matter if we are different services, and our main goal is good patient care.  On a side note, I will mention that the ER resident informed the ER attending of this incident, and there was some communication to the attending of the consulting service about the actions of that resident.  This kind of problem can be documented in your resident evaluation portfolio, and an incidence like this can be very damaging if you are looking to get a competitive fellowship.

Learning From Everyone: I recently observed a situation where the resident asked for a specific setting on the ventilator our patient had just been placed on.  The nurse told the resident that those settings wouldn't be the best and then gave her opinion on what they should be and what is normally done.  The resident got mad and basically dismissed the situation and walked off.  The patient was stable and there was no harm done.  After the resident left I heard the nurses talking. The nurse has over 25 years experience and it turned out was right about the settings.  It made me think about the importance of learning from everyone around us. We have a lot to learn and maintaining a good relationship with our coworkers can make our life easier and our learning more effective.

Nursing Behavior: I thought I had posted this a while ago, but none the less I wanted to relay a story about an interaction I had with a young patient who was complaining of severe nausea and abdominal pain. This particular patient had a fear of needles and was a tough stick. After the nurse tried a few times the patient decided that she would not consent to having blood drawn or receiving anything IV. After expressing her wishes to me, I relayed the message to the resident and we changed the pain and anti-nausea medication to PO. Several minutes later I approached the nurse to confirm she received the order, and she expressed to me that she had gone over our heads and told our attending that the orders should not be switched because the patient was not cooperating and, "squirming like a baby" when attempting to gain IV access. When I explained to her that she needed some type of pain control she, in no uncertain manner, told me that I did not know the situation and that she did.

**Sub-category: Treating patient as a person and not a disease carrier**

On Not Jumping to Conclusions: Several days ago I watched an interesting case unfold, and thought it might be worth sharing-- A teenager came into our ER with the chief complaint of not being able to move his arms and legs since early that morning. The nursing notes stated that he was coming from a juvenile psychiatric facility. Of course, everyone rolled their eyes as they noticed him appear on our trackboard, and no one wanted to pick him up as a patient... he had to be crazy. Everyone assumed that he was faking it, that he probably just wanted some attention or a break from his facility.  After many passed him up for more promising patients, finally one resident went to interview him. When he came back, he told the attending that he truly was not able to make the kid move-- he had tried all the tricks for making patients accidentally cooperate with an exam, and the boy had passed them all. He was able to talk and had full sensation in his extremities, but he could not move them. The attending returned with the same story.  Confused and still not convinced that anything was truly wrong with the kid, they decided to start with a few quick tests to get the ball rolling.  We were all shocked when the boy's potassium came back at 1.3! I felt that this case illustrates an important point, that we must try to be objective when dealing with new problems. Sure, we'll all encounter drug-seekers and other patients who may not be telling the truth, but we cannot make assumptions about other patients who might present similarly. Just because someone has a psychiatric history does not imply that everything they claim is false. I think, in order to provide the best care for patients we must give them all the benefit of the doubt until proven otherwise...

Patient Involved in Illegal Activities: I was working one evening on Fast Track when a pt came in with tooth pain. This patient was reported to have very recently used illegal drugs but rather than judge the patient based on that, the physician I was working with evaluated and treated him as he would anyone else.  I thought it was very professional that he did not write the patient off and pain-seeking and was able to put any judgments aside. He also encouraged the patient to try to stay clean without it coming off as a lecture.  This is the attitude I would also like to maintain in my professional life.

**Sub-category: Using Appropriate language/interaction with a patient/colleague**

STDs: First, I recently had an attending who said "This is what you get for not using a condom" while swabbing a urethra a little more vigorously than what seemed necessary.  While it is true that the pt. must have engaged in some act to get the STD, and he may have been having relations outside of his marriage, we really had no idea...  it could have been his partners doing, in which case not only would he have some emotions to deal with but had just been judged and handled in a less than perfectly respectful way by someone he had come to for help. Second, what do you do when someone in a supposedly monogamous relation asks you how they got an STD?  After finding trich in a wet prep and breaking the news to one pt., she asked if it may have been contracted through some non sexual way since she had only had intercourse with her husband.  I ended up saying that it tends to be sexually transmitted, but it is possible to contract it in other ways.  One physician I spoke with said "if it is an otherwise good relationship, why rock the boat?"  And while there are a few documented instances of STDs being transmitted non sexually, it seems misleading to just tell a pt. that "oh sure it is possible to get that otherways" when that possibility is really, quite small

Arguing the History: Being at XXXs instead of the adult facilities offers an obvious difference with patient interaction.  Parents are the historians and they worse with their kids than they are with themselves.  Frustrating 95% of the time.  Whether it is the parents who smoke around their kid with asthma or the parents who bring in a kid with abdominal pain and he manages to eat a footlong from subway while waiting.  The bottom line is, an objective history and physical must be taken each time and requires you to gain the trust of the parent very quickly.  I worked with an attending of whom with almost every patient we saw, would argue the history of the presenting illness.  This is after I would present and we we go go in to fill in the gaps and finish the workup.  Each time I was thinking, what are we accomplishing by arguing the history?  It is one thing to clarify, another to flat out disagree.  Now, the patient/parents not only do not fully trust the attending, but I get lumped in with that as well.  If life is at risk, then fine, do what you must.  But for gastroenteritis, asthma, URI, etc, where the appearance of the kid will give you just as much info, I cannot reason the point of arguing.

To Scan or To Cut: We had a patient with a classic presentation of an acute appendicitis: 16 y/o kid with periumbilical pain that migrated to mcburney's point with peritoneal signs....CLASSIC! We called the attending surgeon on call and he insisted on obtaining a CT scan prior to surgery. My attending disagreed, but respectfully ordered the scan. The patients father, who happens to be a CRNA at the hospital, arrived at the room and said he preferred a different surgeon. Luckily, this surgeon happened to be in-house already on backup call and was happy to come evaluate the patient for his CRNA colleague. This attending was very confident that this was an acute appendicitis and that the patient should go to the OR without getting a CT scan first. In the ED, we had the fun job of calling the first attending to tell him we had basically gone around him to get the kid to surgery. I felt like my attending did an excellent job walking this very fine line and explaining to the surgeon that we were only acting at the request of the patient's father and that there was no personal issues at play. Ironically, it took almost 4 hours for an OR to become available. Plenty of time to obtain a CT scan.

Patient Care at the Cost of Professionalism: Last week my attending and I saw a 17 yom who c/o periumbilical pain that started 5 hours prior then radiated to this RLQ.  He was febrile, anorexic, and had excruciating abdominal pain every time he went over a bump on his way to the ER.  On exam, he was TTP at McBurney’s point, had rebound tenderness and a +Rovsing sign.  Um, slam dunk.  So my attending calls the surgeon and explains his concern for appendicitis and his opinion that this kid needs to go straight to the OR.  After a few seconds, I hear, “I’m absolutely sure, this kid is classic.  I don’t think we should waste time with a CT.” A few more seconds, “Well, this kid has had 4 CT scans in the past, and I’m uncomfortable exposing him to radiation that he doesn’t need.” My attending’s foot is tapping and he’s starting to look annoyed.  “What about the 1/1000 people you’re killing with unnecessary radiation?!” His voice is getting louder. “Well I can’t do the operation myself, and I can’t force your hand, but I’m concerned this kid may rupture.” I’m thinking, okay, maybe we’re going to compromise.  Then I hear, “Whatever.  If this kid dies because he ruptures waiting for a CT, you can damn well know that I’ll be blaming you and documenting the fact that I recommended surgery NOW!” and he slams the phone down.  Definitely uncomfortable.  So a few minutes later the surgery resident comes down and asks if we’ve drawn a CBC yet.  “What in the hell do you need that for? Is it going to change your management?” The poor resident is just the messenger and explains that her attending wants a CBC.  “Fine, I’ll order it, but this is bulls***.”  Four and a half hours later, the CT confirmed appendicitis, and the kid subsequently went to the OR.  My attending clearly had our patient’s best interests at heart, but I felt his interaction with the surgery team left something to be desired.  I’m posting this story because I think it raises an interesting question.  How should you treat a colleague when you feel he/she is compromising your patient’s care? Are our interactions with our colleagues as important as our interactions with our patients?

Attending Behavior: One of the running jokes in the ER I'm rotating at is that everybody comes in complaining of at least 8/10 pain.  The other day we had a patient who twisted his ankle and was reporting 12/10 pain.  On the way to see the patient, the attending I was with made the obligatory jokes and everyone within earshot had a good laugh about it.  Upon entering the room he proceeded to not-so-gracefully remove the ice bag from the patient's ankle.  He didn't intend any harm, but when the patient winced and made a comment about feeling some pain, the attending was clearly not sympathetic and he said something to the effect that he couldn't be causing any more pain because it was already a 12/10.  I'm not sure if the patient caught the sarcasm or not, but for me the encounter reinforced the importance of respecting your patient.  Even for the patients who aren't really sick or in pain, it's important to at least project the impression that you care, because there's no way to go back once they realize you don't believe them.

Be careful what You Say: I had a personal encounter my first week which, while I don't think was frankly "unprofessional", surely could've been handled better... I was walking to one of my patients' bays when I heard a loud voice calling to me from another bay: "hey, YOU".  I turned to find a young lady in the bed with her mother bedside.  She was obviously ticked-off and continued, "are you Dr. ____ ?" I replied, "No, ma'am, but may I help you with something?"  She said, "We've been here almost 6 hours and haven't seen a doctor yet."  I leaned forward and with a kind smile apologized, saying, "I'm sorry, but it's been a very busy evening and he's got many patients to take care of, I'm sure he'll be by shortly... are you sure there's not something I can get for you in the meantime?"  Again, she stated, "YOU DON'T SEEM TO GET IT, WE'VE BEEN HERE 6 HOURS."  Frustrated, I gently replied, "I'm really sorry, ma'am, but he's been here longer than that today." Immediately I realized that wasn't the best response, but I didn't have much time to ponder before she blew up at me: "WHAT DID YOU SAY? I CAN'T BELIEVE YOU JUST SAID THAT TO ME."  Naturally, I started to apologize, but didn't have the chance before she asked for my name and "supervisor" and so I showed her my badge and told her Dr. ___ was my boss and, hopefully, would be by soon... I turned to leave and immediately retold the encounter to Dr. ____ and apologized saying that I could've handled the situation better.  He laughed and told me she was a frequent-flyer pseudoseizure patient and not to worry, that ANY response would've set her off.  This didn't make me feel much better, and since I've tried to make sure I always try to see the ER from the patient's eyes... and to 'think fast, speak slow".

|  |
| --- |

**Sub-category: Being Respectful to stigmatized populations**

Treating Everyone Equally: There was a 30 y/o white male with herion track marks who came into the ED in a comatose state after overdosing on vicodin, clonidine and his "antidepressant' medication. The young man had to be intubated as he was found to be very hypoxic and covered in his own vomit by the paramedics. After a half hour or so of respiratory support and narcan, the patient was extubated and slowly regained consciousness. Although all of the staff at the hospital worked hard to deliver the best possible care to this patient, I was most impressed by my attending that day. He did not speak down to this patient at all - in fact he took the time to ask his thoughts about what happened and try to uncover the reason for the overdose. He had probably seen hundreds of cases like this before, but he did not seem aggravated by or disdainful of his man's drug abuse and poor decision making. He took the time to clearly and simply explain to the patient what had happened and he even admitted the man for further observation when others had wanted to just send him home.

Drug Seeking: I am at a rural ED, and frequent flyer patients are commonly known amongst the limited number of ED docs.  When choosing patients from the waiting list, most of the physicians would warn me first before I went into the patients' rooms whether or not they were drug seekers.  Part of me was grateful for this warning, but the other part of me regretted this as it ruined my objectivity; I could not help but view the patients as criminals, trying to use me as their enabler.  Was it professional for the doctors to warn a student or unprofessional to take objectivity from my exam?

Delirious: The best instances of professionalism I have seen in the ED are when the OD/intoxicated patients come in delirious and agitated.  Patients interact angrily with the hospital staff.  It sometimes gets worse when the patient’s confusion dissipates and they do not want to be helped or when things do not go as planned in managing these patients.  But in observing the staff in the initial care of these patients, I have noticed how the staff maintains their professionalism by not instinctively becoming agitated and instead are focused on getting the patient better.  These experiences solidify the concept of professionalism for me in that, no matter what the situation is, do your job and maintain respect for the people involved.

Equality: I recently witnessed an unfortunate lapse in professionalism. During an overnight shift in the ED, a Spanish-only speaking family brought their child in with fever and rash. I saw the family then I approached the Attending with the case. The Attending informed me she was busy, so I waited and approached her again. This second time, I was informed that she was "still too busy" to see this "difficult" patient. After continued gentle reminders, four hours later (more than double the wait-time of other equally tiered patients), the Attending agreed to staff this patient. Towards the end of the patient's visit, the father stormed up to me demanding to know whether the family's long wait time was "because we are Mexican?" Equality of patient care is a cornerstone of professionalism. It is not our job to judge, but rather to administer excellent and equal care. I can not speak for the thought process or exact reasons of this Attending. However, the perceived inequality by the family made it real. I hope to never again witness such an infuriating scenario.

**Sub-category: Using inappropriate humor/comments (behind the patient’s back)**

Respecting a patients feelings: On my first ER shift, I was sitting in the nurses station when I overheard one of the interns talking about the patient she had just seen.  She had just examined a homeless women brought in with an old scalp laceration that had become infested with maggots.  She was animatedly describing the smell, the way the maggots had all scattered at the first drop of betadyne, and how she had almost vomited.  Everyone at the station listening to her description was exclaiming in disgust, when I noticed that the patient's bed was situated rather close to the station, and I wondered whether she was able to hear what people were saying. I noticed that she looked extremely miserable, and I didn't like to think that she was humiliated by the comments, in addition to being miserable about the maggots burrowed in her scalp.  As far as I could see, everything was being done to help her - she was taken to the OR to have her scalp properly cleaned, and of course she would receive antibiotics for her laceration, but it seemed like there was no concern on the part of the staff to respect her by not loudly discussing her unusual ailment. In an ER setting, where privacy is pretty hard to come by and people are focused on getting the not-sick patients out the door, how much do people worry about protecting every patient's dignity? When we're tired, irritable, or disgusted, or after we've been treating patients for years and have become somewhat jaded, what can be done to preserve our awareness of patients' feelings?

Lapse in Professionalism: It was an average late afternoon in the ED, more on the slow side, and a middle-aged man had just been brought in by squad in stable condition with mild complaints.  He was initially seen and taken care of appropriately by the staff and attending physician without any issues.  Then his family showed up and accompanied him in the room.  They made a couple of requests to the nursing staff, such as a cool wet towel for the patient and some water, and the nurses seemed to be annoyed by this.  In fact, I overheard them saying, "Oh, it's gonna be one of those patients."  To me this seemed like a lapse in professionalism, because even though the patient was in no acute distress, comfort is also an important concern and should be addressed appropriately.  I guess I can understand that it's hard to keep up your professionalism 100% of the time, especially if it's towards the end of a long shift, etc.  For the most part though, everyone I have worked with has been professional and courteous to all patients even in difficult situations.

Skepticism in Medicine: Maybe it's the naive medical student in me talking but there seems to be a great deal of skepticism in a patient who comes in with pain into the ED.  I understand the need for some skepticism in identifying pain medication seekers but it seems to be blown out of proportion by some medical personnel.  For example, after spending some time interviewing the patient and classifying her pain and researching her previous medical information to find no recent ED visits, I get pulled over by a nurse right outside her room who discusses with me her feelings that her pain is bogus.  I thought this was very unprofessional as she had no rationale for her claims other than "she's being whiny."  In addition, accosting me in front of the patients room to tell me her opinions in plain earshot of the patient and her family was unprofessional.  This is just one incident of what I find to be great skepticism regarding patient's pain or symptoms.  I understand how it is warranted at times but I would rather that it be based on some solid rationale than "gut feeling."

People Are Listening: I know at one point or another we all have frustrations or make a joke that would seem incredibly offensive to someone outside of medicine.  I often hear and used the defense mechanism argument.  Either way, while sitting at a computer reviewing information about a different patient, I sat and listened to an attending use profanity and derogatory comments to a PA regarding one of his patients.  The comments included remarks involving the patients intelligence and the "need for sedation" in order to deal with the patient.  Regardless of how frustrating a patient may be those comments reflected a loss of vision of the bigger picture.  We as physicians have the incredible opportunity to invest and change the course of individuals lives.  Though that may not be in the forefront of minds on a daily basis, it should always be on our minds.  I was disappointed when I heard those comments, and I felt for the patient.  I wondered if the care that patient was receiving was different from a more agreeable or "smarter" person.

Inappropriate Remarks in Patient Care Areas: Similar to most ERs, the one I am currently working in has the nursing/physician workstations in the middle of the patient care area with rooms within earshot of the work area. As everyone knows Columbus has one of the largest Somali populations outside of Somalia (third behind Toronto and Minneapolis). Last week after rooming multiple Somali patients, many of which who were uninsured, a nurse went on a prolonged, loud rant about how she was "sick of paying for these people healthcare, education and lifestyle...for people who are lazy and do not work for a living". Needless to say, this was an inappropriate comment on many levels. Compounding this, it was audible to many patient rooms. The physician I was working with looked quite surprised and embarrassed. He subsequently took the nurse to the side (in an area without patient rooms/traffic) and explained to her in a respectful manner how the comment was inappropriate for the patient-care area. He did not chastise her for the comment or belittle her with his views--he simply explained that it was unprofessional. I though that this was an appropriate way to handle the situation. If no one would have said anything, it would have condoned such activity. Regardless of our viewpoint on such situations, it is never appropriate to voice such views in care areas.

ER Snobs: I've been at XXXs hospital the past 2 weeks and have been having a great experience honestly.  The attendings, residents, nursing and ancillary staff have been fantastic: kind, tolerant, patient, and the teaching has been 1st rate.   My point is that in thinking about writing this I've had a hard time figuring out what would be a good topic. I suppose, and in comparison to some other entries this may be pretty weak, that the most unprofessional thing I've seen to date at the ER is when the doctors/nurses/PCA's make fun of the patients for their coming in for less than urgent chief complaints.  Granted some people knowingly abuse the system cause they know they can get away with it (and I've had patients where its been glaringly clear that they do this), but many others don't know any better and I just found it to be pretty juvenile to sit there and laugh at someone cause they are afraid their kid is sick and in need of help.

Unprofessional Behavior: I am going to be purposely vague on the details because I encountered this poor behavior on several occasions over my clinical years, but this past week I came across a specifically poor example of this behavior. In this situation, a number of health care "professionals" were speaking "harshly" (to put it nicely) about the difficult patients they were caring for. This conversation was in a very public area where many ears could have heard. I wonder who else heard the conversation and what they thought of it, and the "professionals" who took part in it?

Side Conversations in the ED: Something I have noticed on an occasion or two deals with the need to always be aware of the conversations that are going on in any area where a patient may overhear.  There is a central area in the ED where all the computers are located and the majority of the staff do paperwork and "hang out" in between seeing patients.  Patients are walking by this area all the time, and sometimes even walk through the area to get to the other side of the ED.  Some of the conversations I have heard going on in this area have been things that I would not want patients to hear.  They are not HIPAA violations where they are talking about other patients, but are conversations related unentirely to medicine and are about inappropriate subject matter.  By the "letter of the law" these are not in violation of HIPAA, but they are things that I would be embarrassed if a patient overheard.  It's probably a good rule of thumb to not engage in any type of conversation that we would not want a patient or patient's family member to overhear.

Lapse in Professionalism: One of the patients I took care of during my shifts came in with SOB, high-grade fever, cough, hypoxia, tachypnea, tachycardia.  It was fairly obvious he had a pneumonia.  His appearance was somewhat unkempt.  One of the nurses referred to him as a "troll" and another individual called him a "dirtbag."  I felt that this was totally unacceptable conversation at the main ED desk and that the patient was unfairly being judged on his appearance and not on the severity of his illness.

**Sub-category: Criticizing others**

Who’s in charge: During one of my shifts at XX, a trauma was announced with 3 minutes to arrival. The EM team, due to their close proximity to the trauma bays, were the first to arrive. However, it was technically a trauma surgery day to run the trauma. The patient was unstable upon arrival. The vitals were difficult to obtain, and the team was having difficulty stabilizing the patient's airway. It was a very strained situation as any severe trauma is. I noticed a few members of either side grumbling about who should be in charge of the trauma and that they might have managed the situation differently. I think that these members lost sight of the shared goal of everyone in the trauma bay, to stabilize this patient and try to save her life. Instead of grumbling, I think it would be better for these members to either do something useful in the trauma or stand back so that there was less confusion. I do not think that this causes there to be any error in medical care; however, just because it did not happen this time does not mean that these will ever lead to an error or delay in a trauma patient's care. Both sides need to remain professional and realize that it is a stressful situation and that the health, safety, and life of the patient is the primary and shared goal.

**Sub-category: Showing disrespect toward the profession/negative attitudes**

Emergency Room or Non-emergency Room: At the XXX ED, I have had experience observing practice patterns in a rural area with limited resources, but also the eye opening experience of the non-academic setting. In my selected shifts, a current theme has surfaced; the growing problem of access to healthcare. There have been countless patients/families using the ER as their sole primary care resource. The ED physicians, while happy to take care of these patients, often mention that these types of visits actually slow down the flow patient care for those who are in real need urgent/emergent care in the ED.  One ED physician put it best, “this is not what I signed up for 20 years ago.”  This is just one testament to the growing concern that the ED is becoming challenged with even greater strain from the community to deal with primary care concerns in addition to the truly emergency type cases.

**Main Theme: Managing communication challenges with patients and families**

**Sub-category: Handling difficult situations/conversations with patients/families**

Hip Pain: I went to see a 9 month old with the complaint of "hip pain".  Mom and Dad said the child would not crawl or pull up on his crib and had been much fussier and clingier than usual for 2 days.  No trauma, no fever.  Had been to their PCP and Urgent care before coming to ED for x-rays of what was thought to be a dislocated hip.  The exam was completely normal.  I came out of the room to check the x-rays and found bilateral linear tibial fractures in the same place on both legs.  My red flags went up.  The attending and I went back in the room, he chatted with the parents about the history, did a quick exam and told them what the films showed.  He then explained that since the mechanism of injury was unknown in a non-mobile child, laws were in place to protect the child and he would need to order a skeletal survey and head CT.  Through tears, mom voiced understanding.  The MD was at no time judgmental.  He explained what would happen with social work and child protective services and never used an accusatory tone.  The child had been left with a babysitter, but the MD explained that his job was not to find out what had happened - that there were professionals to do this and his only job was to protect the child.  In what could have been a very ugly situation, the parents showed nothing but respect to us b/c they knew we were doing everything for the care of their child.  I was extremely impressed at how well the attending handled this case.  It was hard emotionally for me to handle the magnitude of the situation, the parent's grief and decide if I believed them.  The good news is, I didn't have to make that decision and knew that the child would be safe now.

Amylase again: On a busy evening at XXX, I walked into a room and began the usual routine of obtaining as much information as possible in as short a time as possible.   The workup was relatively straight forward, the gentleman had classic alcoholic pancreatitis.  While walking out of the room and rather sure of my diagnosis, I broadened  the differential to include other life-threatening causes of acute abdominal pain with nausea and vomiting.  I proceeded to present to the attending..  Upon my completion he asked to see the chart and when he saw the name of the patient he became apparently frustrated.  Without saying much he pulled up e-results and looked up the patient.  He went straight to the lab work section and showed me the amylase longitudinal history.  In 2008 this patient had had over 35 amylase levels checked in the XXX. system!!! My attending proceeded to discuss with the patient, his improper use of the ED.  Furthermore we told the patient that he would be treated based off some simple lab results and not because of his symptoms.  To no one's surprise the patient was discharged with a normal amylase levels and no pain medication.  The attending and I entered into a discussion about the misuse of our ED's and the reason was clear, in almost every of the 38 visits to the ED the patient had left with at least a little pain medication. Although our situation I thought was handled well, these patents can present a difficult problem for the ED.  Thoughts>?

Drug Seeker: I saw a patient who complained of back pain, that was acute in onset after lifting a couch the night before. She presented with her friend who also confirmed this story. She also reported to me that she was allergic to ibuprofen, Ultram, naproxen, Tylenol and ASA.  After staffing with my attending, she checked this patient on the database to see what opiate Rx she had received in the last year.  In the last 52 weeks, the patient had 51 Rx for opiates. After finding convincing evidence of drug seeking behavior, my attending didn't complain at all about pts like this, but taught me the appropriate way to approach them, turning it into a teaching session. Instead of outright accusing her of being a drug seeker, my attending let the patient tell her the story, and asked her if she had received any meds for pain in the last year, giving her a chance to tell the truth.  When the patient denied any pain meds, she then proceeded to show her the proof she had. Even after being caught red-handed, my attending continued to offer her options for pain control, just without opiates. She never told the pt that she didn't believe her pain, but just told her that opiates was not the way she was going to help her. Pt eventually refused and left, but I thought my attending handled this situation in a professional way, both to the pt and me.

Patient privacy: A 17 yo F is brought to the ED by her mother with a month of nausea and vomiting and was unsure of her LNMP. Her mother was present throughout the history, physical, and rest of the visit. Not surprisingly, the patient's b-hCG turned out to be positive. This news was relayed to the patient with her mother present. A pelvic exam (the patient's first) and transvaginal US were then performed to confirm intrauterine pregnancy. In this case neither the patient nor the mother had any problem with the way the visit was handled. However, I can imagine that this may differ from patient to patient. Thoughts on patient privacy in general? Relaying results? Peds vs. adults? Procedures? Family members, friends, etc.? And practically speaking, if a guest is asked to leave a room with a curtain, where must they be sent so as not to overhear what is being discussed?

Handling a Delicate Situation: I was witness to a very challenging situation last week.  Late in the evening, a level one trauma was called and as the ER was incredibly busy, several available people including me rushed to conduct the primary and secondary surveys and stabilize the patient.  The patient, a 16 year old female involved in an MVA, was conscious despite multiple wounds.   We received the news that a guardrail had impaled the passenger (the patient's mother) on the spot, and she expired before the EMS could arrive.  The news was initially conveyed quietly to the attending, who stepped away from the scene momentarily and informed select staff to keep the news from the patient for the time being. The patient reported that she had been driving with her mother and noted another car weaving in the oncoming lane. She tried to avoid a collision, but in doing so speared a guardrail head on.  The patient was disoriented at the scene, but managed to call her father who called the highway patrol.  She was brought to XXX and had left her mother awaiting the next ambulance to take her to an adult trauma center. The father & brother arrived at XXX, and the father said that he was going to leave the brother & go to whichever hospital her mother was being taken to.  The attending told him that as soon as we knew where she was taken, we would inform him and he could go to be with his wife.  About 20 minutes later, the father was called outside while the brother stayed with his sister and offered support at the bedside.  He was taken to an empty room, made to have a seat, and the news was conveyed honestly and frankly.  He was obviously distraught, but managed to recover his composure.  He was asked whether he would like his son to know now or later, and if he would like to give him the news or whether he would prefer that healthcare staff did.  He chose to give him the news himself, and called his son into the room.  The son fainted upon hearing the news, and had to be revived. He was inconsolable. A chaplain was present at the scene and offered comfort, and spoke with the boy alone upon the father’s request.  The news was withheld from the patient upon the family’s request. Personally, I think the situation was handled well and that timing is of paramount importance when delivering bad news.  I also think that the distribution of information was handled well, with a quiet, isolated place where the bad news was conveyed and a chaplain and other support resources on hand to help them deal with the immediate grief.  But for the purposes of discussion: -Is it right to withhold important information in order to make what you need to do go smoothly, or to lie and say that you have not received any news yet?  -Was it fair to the father to put him in the situation where he had to decide whether his children would hear about their mother’s condition when he had just seen his only daughter bleeding and naked on a trauma bed?    -Was it right to continue to tell the patient when she asked about her mother  that we were awaiting news from the adult trauma center while her brother and father were in the other room dealing with the news

An Approach to the Addicted Patient: A 43 yo female with Hx of multiple GU diseases including nephrolithiasis x 4 and recurrent UTI/pyelonephritis presented with 3 day hx of vomiting and 8/10 persistent, bilateral flank pain that radiated to the groin. Pt. was seen in ED one month prior and CT scan demonstrated several 2-3mm nonobstructing calculi in one kidney. A UA, Urine dip, CHEM7 and CBC were all unremarkable. After checking OARRS, we discovered that the patient had been to multiple EDs over the past year filling 32 different controlled substance prescriptions at 20 different pharmacies. I thought the approach of the ED attending in this case was commendable. Since this is a very common scenario which I am sure many of us will encounter in the future, I thought I would address it. He first approached the patient and revealed the information he had discovered. The patient then became defensive and hostile, but eventually confessed to filling the multiple prescriptions. Rather than reciprocating the patient’s behavior, the physician remained cool and collected conveying a sense of compassion and empathy towards the patient. He told her that he believed that she did have an organic cause for her pain, but given the history of opioid dependence, he would not prescribe any controlled substances but would prescribe anti-nausea and non-narcotic pain medications as desired. The patient then agreed to this plan of care. There are a few points that I think were important when dealing with the drug seeking patient: maintaining a nonconfrontational approach and avoiding an adversarial tone, maintaining respect for the patient avoiding a punitive attitude and continuing to provide high-quality, compassionate care.

Discussing DNR status: I had a patient come in over the weekend who was short of breath, and had a history of lung cancer, not exactly a particularly complicated differential diagnosis right?  When I stepped in the room to see the patient it was immediately evident that the triage nurse and the patient were not fully aware of the severity of this poor woman's disease.  After conducting my H&P I pulled up her most recent imaging, CTs from 2 months prior, which revealed metastatic disease in nearly every vital organ not to mention near complete involvement of her thoracic cavity.  This woman was short of breath because she had almost no normal lung parenchyma.  When I presented the patient to the attending he immediately called the woman's oncologist who stated that they had touched upon hospice care in the past but had not discussed it or DNR status definitively.  My attending handled the ensuing conversation expertly but I felt sorry for both he and the woman for having to discuss this at all, especially under the circumstance where her respiratory status was decompensating so quickly.  Knowing that her scans 2 months previously clearly indicated the inevitable terminal nature of her condition I believe that DNR status should have been addressed definitively by her oncologist at that time.  While these are not pleasant topics to discuss I fell that we owe it to our patients to include these topics as part of the "treatment" plan in situations like this.

IUD Removal Followed By Miscarriage: We had a woman in the ED the other day who had undergone an IUD removal by her OB.  She came in for bleeding and cramping after the procedure.  Her Quant B-HCG was <2 and we couldn't find any products of conception on U/S. My attending informed her that she had just undergone a miscarriage.  He then went into what sounded like a very traditional talk on spontaneous abortion.  I was surprised to hear him going that route, since this was likely an unwanted pregnancy.  But then I realized it's a very smart thing to operate under the assumption that she really had wanted this baby and that the miscarriage came as bad news.  At least it's better than assuming that she'd be happy to hear about the miscarriage when if fact she was not. It's not easy to know what a patient perceives as "bad" news.  I learned that if we're not sure, we're probably better off treating it like bad news until proven otherwise.

Another Code Well-done, Although Unsuccessful: Recently, at the XXX's ED we had a 5 year old girl presenting in full code.  The situation was complicated by the fact that there were two sets of parents - foster parents and biological parents.  The foster mother arrived with the patient, and she stood in the room watching the efforts and crying, with support from social and pastoral care workers.  All this time, the code was running smoothly, with no one making the foster mother feel as though she were in the way or not welcome.  When the foster father arrived, he entered the room and held the girl's still hand in silence for a few moments, and the team worked smoothly around him. When the biological parents arrived the patient was just being pronounced dead, the code having been run for an extra long duration of 1 hour due to a briefly regained pulse.  The biological father entered the room, said that XXX's was the reason his child was dead and he was going to sue, and left the room to repeat this to everyone nearby.  The biological mother remained in the room and began to sob.  Her husband having left her alone there, she was immediately hugged by the nearest nurse and allowed to cry on her shoulder.  The attending physician remained for several minutes until the mother was ready to know what had happened, and explained the situation with compassion.  While the mother stayed in the room to be with the child for a while, the attending went into the hall to repeat this for the foster family and the biological father, helping the foster family not to blame themselves wrongly, and calmly absorbing the continued verbal attacks of the biological father.  The next day, the attending had come in off hours to investigate the results of the patient's pending cultures.  He saw me there on my next shift and stopped to discuss why the girl had died, why the code had finally been called.  He answered my questions and showed me the test results that had come back in the ensuing hours to give further explanation for the cause of her death.  All in all, I thought it was an exceptionally difficult code situation handled with skill and compassion, even if the patient could not be saved.

Narcotic Seeking Patients: I recently saw a case where a patient presented for a migraine that would not go away despite his normal prescribed treatment regimen. He looked like a perfectly trustworthy guy and seemed well educated and interested in ultimately controlling his headaches. However, when I looked up the patient's past records it showed many visits for the same complaint where it seemed he only wanted narcotic treatment. There were several notes from ED attendings explaining that he frequently visited then left immediately after getting a pain med shot, and one attending had records from the Board of Pharmacy showing a pattern of narcotic seeking from multiple institutions and prescribers.  The attending and I spoke with the patient, and I was extremely impressed by the approach the attending had at being firm about the situation while still showing concern for the patient's condition. The patient ended up leaving AMA after receiving a small dose of pain medication anyways, but the professional manner the attending employed was pretty inspiring.

Pain Medications: During one of my fast-track shift at XXX, I saw quite a few of slip and fall complaints due to the recent ice storm we have experienced. Interestingly, there were two patients came in on separate visits about 2 hours apart with similar complaints of fall and back pain. The attending pulled out the Ohio narcotic prescription registry and found out that both patients received his and her pain medication from the same doctor from outside area of Columbus. Later, we also found that the two patients live in the same address! Although, there was always a slim possibility that their pain is legitimate, the behavior is certainly suspicious. I thought the attending handled it very professionally by listening to the complaints before performing the examination, then directly but politely confronted the patients that he will not prescribe them any narcotics today by focusing only on the clinical presentation. As for the suspicious of narcotic seeking behavior, we made an objective note on the record of our finding from the registry.

An Excellent Example: My attending and I saw a patient yesterday at XXX’s Hospital ED that really tried our patience and professionalism. The pt was a 4 y/o wm who had been seen in the ED before Christmas for a rash that was dx'd as Chickenpox. Well, 2 months later and the pt still has the rash. The pt's mother calls EMS to bring the pt in because she has no transportation. I go in to see the pt who is obese and has to be picked up by mom to put him on the examination table. The rash is  maculopapular and spread all over his entire body and he is visibly scratching at it while I am examining him. (Oh, and in the ED's defense the rash had started on the pt's back which not too many rashes do except Chicken pox). So I go to relate the hx to the attending and she is visibly upset that the pt's mother waited two months to do something about her son's condition, that WICC is involved for the obesity, and that they used valuable resources to get here when it clearly does not sound like anything life threatening (i.e. measles, hives, etc). But when the attending went into the room I saw her put aside her feelings and calmly explain to mom that this is not a life threatening rash and we therefore could not do anything further from an ER standpoint, and that it is important to have regular follow-up with the child's PCP so that her son doesn't have to suffer unnecessarily. (In case you're wondering the attending thinks the rash was keratosis pilaris). The mother didn't look happy but she took everything in stride and asked for a bus pass which the attending agreed to. The attending stayed cool throughout the entire encounter even with the pt playing with the switches on the bed, the otoscope on the wall and throwing himself on the ground. Later, we found out that the mother then made a big scene in the waiting room waiting for the bus and was upset when denied a cab pass and had to be escorted out by security. I think that's even more evidence of just how professional my attending was that a difficult mother did not erupt in the room even when she was clearly unhappy then.

Approach to the RX Seeking Patient: observed what I thought was a very effective method of dealing with a frequent patient to the ER who presented with vague lower back pain which, upon further exam, was decided to be musculoskeletal with no neurologic symptoms. The attending first explained to the patient what our suspected diagnosis was and offered his treatment plan of NSAIDS and a muscle relaxation. The patient was visibly unhappy with this plan of action and pushed for some sort of narcotics for "unbearable" pain. At this point, the attending went to the computer and checked the online pharmacy resource and learned that the pt. had filled a script for 60 Vicodins less than one week earlier. He returned to the patient's room, informed him of our knowledge and firmly restated what prescriptions he thought were prudent considering the patient's current status. I was impressed with the non-negotiative attitude of the attending in this encounter and find it can be a very effective approach.

Dealing Professionally with Sensitive Topics: Most times I can understand that ER work is hard and busy and sometimes there are patients that annoy you, but I think that most ER docs do a good job of dealing with patients that they don't want in a professional manner.  However, one night I was working at the ED and a patient comes in with her boyfriend and I told the doc, after performing the pelvic and doing cultures, that I thought she had PID.  Instead of also talking with her and discussing the situation calmly, this doctor walked in the room, demanded that she tell him if the boyfriend was her only sexual partner, and told the guy that one or the other had gotten an STD and he needed to go get tested and treated or she was going to get this disease again.  While I think that this discussion about whether the male needs to get tested and treated needs to happen, I don't think that this was the most professional way of handling the situation.

When to Evaluate: Working in the XXX’s ED, one of the most common complaints I have witnessed are when parents bring their child into the ED after seeing their PCP and being told their child has a viral illness.  At this point, we're put in the position of either allaying the parents' fears by running further diagnostics(and increasing their bill), or sending the parents home unhappy and usually still concerned by echoing the sentiments of their PCP.  While reassurance can be by far much more difficult and less appreciated, it is definitely in the best interest of the patient and family, even if they don't appreciate it at the time

Narcotic Seeking Patient: Certain patients are referred to as frequent flyers for pain medications. Some even make regular visits to each of the XXX system emergency departments until one of them stops seeing them and they move on to the next one. It is surprising to meet someone for the first time and they tell one story of what seems like new acute onset pain and then you go to present to your attending and they hand you the Ohio automated prescribing system printout which tells a completely different story. The ED physicians I have worked with have done a great job at interacting with these patients professionally, compassionately, and directly. They make it clear what tests will be ordered given the patient presentation and the next steps depending on what each test shows. They make it clear up front in a kind, direct manner that narcotic medications will not be prescribed if imaging and blood work does not help us find a cause for the pain which will be treated and that pain medications will only be used in the interim for control.

**Sub-category: Communicating in a caring and compassionate way**

Poor Professionalism: Everyone has encountered the patient that does not understand the severity of their problems or does not have the resources to have proper health care. During a busy night shift when all the beds were full and people were waiting in triage I saw a patient who came in with a simple complaint that led to a serious problem. She was an elderly female with 2-3 wks abd pain with nausea and vomiting with severe cardiac risk factors. On further questioning she related sx of chest pain and sob with exertion. I went to tell the attending that the patient had significant cardiac risk factors with a concerning history and that the patient will most likely need to be admitted for more testing. The attending went to see the patient to confirm the severity of the situation. He agreed and wanted to admit the patient. The patient did not want to be admitted because she just wanted her abd pain to be fixed so she could go home to take care of her grandchildren. The attending tried to explain that the abd pain was not as concerning as her cardiac history and that she would need to be admitted to rule out a MI. The patient continued to refuse admission and asked repeatedly if we could just fix her abd pain. The attending began to get frustrated. He then went on to tell the patient that if she went home she would come back in full cardiac arrest and die. The patient began to tear and at the end still went home against medical advice. I think that lapse in professionalism occurred when the attending tried to scare the patient into staying in the hospital and then failed to educate the patient with the proper information.

Exemplary Compassion for Patients and Their Families: During a clerkship shift at XXX, my attending was informed of an incoming patient arriving by EMS with a CC of "unresponsive". Upon the patient's arrival, initial evaluation made it very clear the patient had sustained a massive stroke. CT confirmed a large pontine hemorrhagic stroke that certainly posed a risk to the patient's life. Amidst the discovery of the severity of the situation and following the intubation and stabilization of the patient, my attending made it an priority to seek out the patient's family members who were anxiously awaiting any news. I was very impressed with the consideration and empathy the attending displayed to the patient's family. Shortly thereafter, a neurosurgery resident arrived to assess the patient. He, too, recognized the importance of seeking consultation with family members as early as appropriate. Though this clearly was the "right thing to do", I was reminded that qualities like COMPASSION and EMPATHY for patients and their families are tenets of medical professionalism. It is often easy to get carried away and distracted with the responsibilities of our jobs as medical professionals that we must take caution to not neglect to share a moment with a family member who may lose a husband, brother, father, etc.

**Sub-category: Communicating with angry/resistant patients or families**

Side Effects: I recently had an interesting experience during the ER visit of a young boy with epilepsy. This unfortunate little guy has a severe seizure disorder which has required escalating dosages of medication over the last two years.  At his last outpatient Neurology visit, the dosage was increased yet again to deal with breakthrough seizures.  However, he was now near the upper limit of dosing and was beginning to experience some serious side effects and that led to his ultimate ER trip. During our workup we spoke with the Neurology fellow who recommended decreasing the dosage slightly to still provide seizure protection with lessened side effects and to follow up for his regularly scheduled Neurology appointment.  When we shared this plan with his mother she became increasingly upset with us, demanding that we either get him an appointment at the Neurology clinic for the next week (his was scheduled for the beginning of August) or that we admit her son to the Neurology service and get a Neurologist to see him right away. I am sure that everyone is aware of the serious shortage of pediatric specialists in the US and Neurology is certainly not an exception.  Our dilemma was how to determine if his needs were so great that it justified bumping a different neurology patient, who had been waiting patiently for at least 6 months for an appointment, because our patient's mother complained louder and longer.  It is difficult to determine how to appropriately allocate resources in a fair way when you have more patients than you can handle and they all greatly need your help. In the end we convinced her to keep her original appointment after some careful and gentle insistence.

Stages of grief : A couple of weeks ago, I had been following a very pleasant women in the ED who had non small cell lung cancer and had recently undergone a colonoscopy to look for other masses.  She presented with diffuse, severe abdominal pain ever since the procedure and we were concerned that she might have a perforate bowel.  No air was seen on the upright CXR and we wanted to proceed to an abdominal CT to further evaluate her pain, but she declined stating that she had received way too many tests recently and didn't want the radiation exposure.  During her workup, we also discovered that her platelet count was 19,000!  When we told her she was going to have to be admitted to the James for further workup and treatment, she got angry with the staff and began saying that she wanted to go back to her hometown hospital where they would treat her better than we had.  While we waited for a room assignment, the complaints grew louder and she turned into a very difficult patient.  The attending and I went into to try and calm her and I was very impressed at how he handled the situation.  He remained calm and tried to answer all their questions and was not at all defensive when the patient and her husband blamed the hospital for trying to keep her against her will.  After we left the room, the attending asked me what I thought was really fueling her anger and we discussed that she is going through one of the famous stages of grief.  I think this was a good example of trying to put yourself in your patient's shoes before judging their behavior.  It would have been very easy to get angry at this patient who was complaining about every aspect of her visit and try to ignore her until she was admitted, but instead the attending went into the room voluntarily and let her vent for awhile, all the while remaining professional and supportive despite her attacks.  I think it is very hard not to get swept up in the emotion of a busy ED and a good lesson to take a step back sometimes and try to imagine yourself in the patient's position and how you might react.

Personal assumptions affecting patient care: A 49 yo male with a history of CAD and an abnormal stress test within the last year was brought to the ED by EMS for midsternal chest pressure, diaphoresis, and trembling.  His presentation was complicated by possible narcotic withdrawal, as he had not taken oxycontin in 3 days.  He had recently been hospitalized for a seizure.  When his previous records were reviewed, it was noted that his prior hospitalization for seizure was more likely a drug overdose, the record stated that upon EMS arrival the patient's daughter was giving her father numerous tablets of oxycontin, stating that her father needed his pills.  When the attending asked the patient about the circumstances of that admission, including why the patient was given narcan, the patient stated that every time he has a seizure he receives narcan and he has never overdosed on his narcotics.  He became very upset that the record stated that his daughter had been feeding him pills, at which time he called his daughter to have her explain the situation.  The daughter called back to the ED and began yelling at the unit clerk about the accusations that were being made towards her and her father.  After the phone call the unit clerk was discussing the situation with the nurse, of which the patient overheard.  He became even more upset and began stating that was was going to leave and go to another hospital because he did not like the way he and his daughter were being treated.  He felt that the nursing staff was very rude to him because they felt he was an addict, and was additionally upset that a former hospital employee would be treated that way.  At this time the attending physician  went to talk with the patient.  He explained that we were not accusing him of overdosing on narcotics, we were only trying to clarify the situation.  Also, he explained to the patient and the daughter that we were not treating him as a drug addict, as we had already given him pain medicine to alleviate his symptoms.  Eventually he was able to calm both the patient and the daughter and the patient remained in the ED for evaluation.I feel the attending physician handled the situation very well.  However, it seems that the support staff let their personal assumptions about the patient affect his care and he was not treated with the respect and compassion that patients deserve.  I think it is important that we become aware of our opinions and assumptions and the way they can affect patient care, and that we work to push those assumptions aside to focus on the current situation.

My Most Difficult Patient Ever: On my first overnight shift at XXX, I encountered a very hostile patient.  He came in complaining of lower leg cellulitis and suture removal for a previous arm laceration.  This was a patient you could tell had been in many previous fights due to various wounds at different stages of healing.  I went into the room by myself and immediately I knew there was something dangerous about this patient.  He became very frustrated and angry at me when I asked my usual bag of simple questions.  He couldn't understand why I didn't know everything about him already and why I couldn't just take out the sutures immediately and cure his cellulitis.  Not two minutes into my history he sat up in bed in an intimidating manner, as if ready to hit me.  Usually I am pretty good handling difficult patients, but at that point I had had enough and I left the room.  A few minutes later he started cussing out the nurses and tech's, forcing a slew of cops to come over and keep things settled.  At one point he even threatened to come back and shoot the nurses with tommyguns, a term I think I have only heard in the movies. Everyone in the ER just wanted the cops to kick this guy out (or arrest him) without being treated, including myself.  Why should we treat this guy who is clearly dangerous and threatening the staff? But my attending handled the situation with great poise.  With the cops closely watching, he removed the sutures and explained that the only way to treat the cellulitis was to take the full prescription of antiobiotics.  My attending was firm and confident, showing no signs of fear or intimidation, was never argumentative, and never responded to the patient when the patient continued making angry remarks at the hospital staff.  It was professionalism and commitment to duty put to the test, and something I will never forget.  p.s. If any of you have any thoughts on this, let me know.  We discharged the patient (as quickly as possible), but I was wondering if we should have pink slipped him.  Suicidal ideation gets a quick pink slip, but what about homicidal threats?  This guy was clearly telling us that he could come back and shoot up the nursing staff with tommyguns; I couldn't tell if he was joking or not.  Regardless, should we have pink slipped this guy on the basis of homicidal ideation?

Professionalism: We had a man brought in who was in police custody after crashing his car in an attempt to avoid arrest.  He had a few cuts and bruises, but otherwise appeared intact.  He was extremely combative and aggressive, shouting obscenities and at one point attempted to attack a nurse.  The doc was professional the entire time - she didn't respond to his antagonism, nor did she let him push her around.  She was able to maintain control of the situation (as best she could, anyway) without resorting to unprofessional or rude behavior.

Professionalism: I experienced an appropriate professional reaction to an unreasonable and irate patient.   A patient who demanded an MRI for no reason other than she thought it was what she needed, was getting agitated about not getting what she wanted when every appropriate diagnostic test came up normal.  The attending, instead of blowing her off or strong arming her sat down and assured her that she would be taken care of, explored exactly why she felt she needed an MRI, and convinced her that she should try our treatment and then if she wasn’t feeling better, we were here and ready to see her in 3-4 days.

Family Members: On my last shift we had a 30 year old 400 pound female brought in by squad for loss of consciousness who then coded. She had been in the ER several days ago and had been cleared for a complaint of shortness of breath by another ER doctor. By the time she arrived this time, there was little we could do and the patient expired within about 30 minutes. The family was extremely angry and yelling at the doctor that we let her die because we did not catch the problem. They demanded to talk to the doctor that let her leave. When we told them that wasn't possible and that she had been cleared the last time she was here they didn't want to hear it. Clearly grieving, they were angry and rude, and eventually we had to call security. Rather then get angry or defensive, the doctor I was working with was very understanding and did not take offense. The family came in later and thanked her for her kindness.

**Major Theme: Demonstrating responsibility, pride, knowledge, and thoroughness**

**Sub-category: Displaying responsibility, honesty, and integrity**

End of shift: A week ago, I had seen a patient in the ED at XXX's who had been complaining of back pain and overall malaise.  She had been non-compliant with all of her lupus medications.  After doing my H&P, I told my attending that I was ready to present my patient.  She had some other patients to see and told me she'd be ready to hear the story soon (this was an hour and fifteen minutes before the end of her shift so I thought I'd definitely be able to present my patient and work her up).  Just before her shift ended an hour later, she told me that she couldn't pick up my patient and I'd have to present her to one of the incoming attending docs.  Obviously, this delayed treatment even further.  Although my patient's needs did not require urgent care, I was a little disappointed that she wasn't getting more attention.  At least this situation does not seem to happen all that often and is dependent upon the severity of illnesses of the other patients in the ED, traumas, etc.

Ethics of Procedures: I recently had an encounter regarding the ethics of patient informed consent for procedures.  The discussion revolved around how much information to give the patient when advising them for or against procedures.  I was particularly disturbed by the comment by the attending that he will tailor the number and degree of risks of a procedure based on how important the test is.  The part that is disturbing is that he states he would minimize the risks if he felt like the procedure was really important and maximize the risks if he felt the procedure was not needed.  He also stated that he really only presents the risks/benefits equally when he is on the fence (50/50) as to whether the test will help with diagnosis.  I really feel that if the patient is able to make an informed decision, they should be able to also know all the relevant risks and benefits.  If the benefits greatly outweigh the risks, that is fine to say, but the patient should still know the risks, and they shouldn't be minimized. The part that is disturbing is that it seems very paternalistic, whereas throughout all of our medical school so far has been emphasizing the importance of patient autonomy.  I guess I didn't realize how much of an influence PCM has had on my perspective of patient autonomy and how important I think it is, especially in this day and age when we are faced with so many possibilities of malpractice. Does anyone have any input on to resolution of paternalistism v. patient autonomy (and patient comprehension level)?

Breaking good news: On Tuesday evening, a 49 y/o female presented to the XXX ER with right-sided hip pain. She had a few drinks earlier in the night and had tripped and fallen on her right side.  She reported 7/10 pain and had mild tenderness on exam.  Our clinical suspicion for serious injury was low, but we ordered a plain film of her pelvis.  We were surprised when the radiologist's impression came back as, "acute fracture of the right hip."  The attending notified the family and preparations were made to admit the patient.  But later, as I was talking with the attending, we both admitted our surprise at the diagnosis; her presenting symptoms just didn't seem to fit.  So we looked up the image of her plain film and failed to see an obvious fracture.  A call to the radiologist revealed his mistake.  He had meant to type, "NO acute fracture of the right hip."  The attending walked straight over to the patient's room and explained the mistake and offered his apologies.  I had expected the family to be angry, but instead they expressed their relief and thanked him for explaining everything. I think this case illustrates two important points: 1) If lab results don't seem right, you need to follow up! 2) Patient's will appreciate it if you are up-front and apologize when mistakes are made.

Consults: As everyone has experienced by now, sometimes getting consultants to see patients in a timely manner can be difficult. Recently we had a patient who came in with head, neck and back trauma as well as trauma to the extremities. In trying to decide what needed to be done, we felt that the injuries as we had evaluated them warranted a consult to Orthopaedics (note, this is being written by someone entering that field). Our consultant, after we talked to him, indicated that there were a couple of points of history that he felt should first be run through Neurosurg, then he would see the patient afterward. So we called neurosurg. Interestingly, the resident on call had a similar attitude- have the other guy see it first, then I'll come down. We went back and forth for 30 minutes or so before one of them finally conceded and came down. And here I was thinking that I went to medical school to learn how to take care of patients, not avoid them.

Scary Consult: Earlier this week, we had a little kid come in with new-onset anisocoria (unequal pupil size). He was young enough that a full neuro exam was difficult (also, I think he had like 6 words in his vocabulary), so we were fairly concerned that we might be missing something that needed emergent care, possibly in the OR. We called Ophtho to get things moving along, and were told "its most likely just physiologic. Call us after you get the CT." This left us scrambling through the internet trying to figure out how to properly assess the kid and figure out if it was physiologic.  As we all know, the diagnosis of exclusion is not what you want to try to confirm in any situation, but it’s particularly frustrating when you're dealing with potentially vision- and life-threatening conditions.  It did end up being physiologic, but I think that the situation deserved more attention than it got.

Professionalism: I had an experience earlier in the rotation - one of the attendings seems to disappear for 30 minute intervals fairly often during his shift; none of the staff are aware of his whereabouts. Although the ED I am in is low volume, and this attending does have an administrative position within the hospital, I am unsure what is the appropriate time balance for an ED attending to actually be in the ED.

Unnecessary Tests: On one of my shifts, the attending that I was working with was very quick to order a battery of tests on all patients - regardless of indication.  One particular patient was ordered a head CT although his story was not concerning for any serious pathology.  When I inquired why a head CT was being done, the attending responded "Well, he came to the ER.  We have to do something."  This struck me as not only a waste of resources, but it was also exposing the patient to unnecessary radiation.  I understand the attending's wish to satisfy the patient and to avoid missing anything, but in several instances that night (and in this one in particular) I felt that he was doing the patient more harm than good with the unnecessary tests.

Hospital Shopping: I have had many patients this month that have demanded to be transferred to our facility because the other hospital wasn't "doing enough".  We had a patient's family that was upset because they were at another ED for 2 hours and they felt as if they didn't "do anything".  I understand that EDs have long waiting times but it seems as if most patients don't.  When this particular family got to out ED they had to wait again and the attending decided to do unnecessary treatment to keep the family happy.  I felt as if this just perpetuated the cycle of families feeling like our facility "does more" because we do when they come unhappy from other places.

Giving Parents What They Want: Functional abdominal pain the XXX's ED is common, but parents don't like to accept this as an answer.  It is often easier to given parents something more concrete to focus on.  Almost always, these kids get an AAS, which show some amount of stool in the colon.  Attendings often take the angle, "Your child has poop everywhere in the colon; here is some Mirilax"  Sure, some of these kids probably are constipated, but I feel like half the time I am starting to fixate these kids on their bowel movements even though the amount of stool is physiologic and likely unrelated to the problem.  It's a heck of a lot easier to blame things on constipation, then to start forcing the pt and parent to deal, but I think this needs to be done more often then it should.  At the very least, the constipation theory needs to be hedged more than it is.

**Sub-category: Thoroughly investigating patients’ problems**

Professionalism: We had a patient who came in with a complaint of chest pain. This patient has been seen here for a total of 28 times with the same complaint. Nothing has ever been shown on his work-up an the only thing that has ever made it better is dilaudid in the past. Once agan we had to do an initial evaluation for and ACS, but since hs EKG was completely unchanged since I saw him in the ED 1 week prior, he was not rushed back for his full work-up right away. I thought this was appropriate because of his past history, no change on EKG, and no change in his presenting symptoms from all his previous visits. Any thoughts?

Suicidal Ideation: If someone says they want to kill themselves to a friend and changes their story after they get to the ER, it can be a difficult situation.  This is exactly the scenario presented a few weeks ago.  The friend was told over the phone so she called the patient’s brother and the he went over to the patient’s house to investigate.  He was banging on the door for 30min before she let him in and by that time the EMS had arrived.  Luckily they arrived in time.  She told her brother at the time that she just didn’t hear the door or her cell phone.  After getting to the ER, we learned she was a nurse that works in the hospital and she seemed to change her story right after hitting the door.  The doctor had a difficult decision to make but ended up admitting her for her own safety, and I tend to agree.  The nurse expressed a concern over losing her job if she was pink-slipped, but if she went home and killed herself, I thought that outweighed the situation.

Too Quick To Dismiss: I had a patient who was a poor historian present with a complaint of vaginal bleeding.  She had had a hysterectomy for fibroids so that couldn't be good.  Later during the history taking, the pt remembered that she had been treated for cervical cancer in the past, so I thought that it was possible that she had disease recurrence.  On spec exam I saw an ugly, bleeding mass.  I told the attending what I saw.  In the attending's mind, this was no longer an acute problem.  The attdg ordered a UA (which was nml) and sent the pt home with a number for GYN follow up.  Although the mass wasn't something that could be dealt with immediately, I wondered if it wasn't worth checking a CBC to r/o anemia or imaging her abdomen since she complained of abd and back pain as well.  Clearly the pt had a lapse in follow-up since her original dx of cervical cancer, and I questioned how quickly she would follow-up.  I wondered if there was anything more that the ED could do for this patient.  Was the attdg too quick to dismiss the patient's concerns?  I'm sure during this month, we've all seen patients who come in with one complaint but something else grabs out attention.  We want to pursue it, but the attdg does not. I did call the patient and stress that she follow-up in a timely fashion.  I followed up with her again, and she had the lesion biopsied.  She appreciated that I called her and requested that I call again to find out what the bx showed.

**Sub-category: Striving toward excellence**

House Fire: I'm not sure how many of us watch the new these days (seems like it's all bad stuff), but if you happened to catch a new program last friday or over the weekend, then you may know what I'm about to talk about.  I was assigned to EMS 17 for my medic ride last friday.  The day was going along as normal - listening to the occasional alert of fire runs over the intercom while chatting in the break room - until about 11:00am.  This time, when the alert sounded, everyone took off at a full speed sprint.  I wasn't sure what to do other than try to catch up as best I could.  When we made it into the cab I was able to finally hear what the voice over the intercom was saying....we were being called to a house fire and there was a 4 year old boy still trapped inside.  By the time we got to the scene, my adrenaline was sky-rocketing.  Someone threw me a pair of gloves and told me to remain by the stretcher so that they would know where I was at all times.  Neighbors began accumulating outside in small groups and it wasn’t long before news cameras set up shop on front lawns all around the house.  Minutes seemed to pass slowly, but when a firefighter emerged with the boy, we jumped into the ambulance and raced to XXXs hospital.  Despite the best efforts of everyone at the scene and at the hospital, we were unable to revive the boy.  Obviously, the entire event was highly emotional.  I've never been on that side of an emergency and I give enormous credit to all firefighters and medics that I worked with for having the strength to act professionally and get the job done despite the chaos, the yelling and the cameras in their face.

Good Example of Professionalism: We had an extremely busy night few days ago with ER filled to capacity (including hallways) and plenty of people in the waiting room. Despite that, I was amazed by the professionalism showed by both attendings and senior resident. They remained calm, and listened to and tried to accommodate all complaints/needs of patients/nurses. They never seemed angry or annoyed. They did not seem to be rushed in patient encounter despite busy ER, and listened to patients very carefully. Overall, I think they provided a very good example of professionalism in a pressured environment.

Competency in the ED: From what I have seen the ED, it is clear that emergency medicine doctors must be competent in a wide range of every specialty. In addition to integrity, altruism, compassion, respect, and the other tenets of professionalism, competency is equally important. As one of my attendings told me during this rotation, they only get to a quick snapshot into someone's medical condition; therefore, while an internist may search for reasons to keep someone in the hospital, ED physicians work in reverse and find reasons to let them go home. They must think through a wide differential for every patient because these people may not have a PCP or get any follow up. Though many symptoms may have a benign etiology, the nature of the ED is to rule out the potentially life-threatening ones. Missing one costly, but necessary, test can be a matter of life and death and they may not get a second chance. They must also know when it is appropriate to consult, pink slip, admit, arrange follow up, or send home.

Judicious Testing: Prior to the rotation, I've heard a lot about how the ER "just orders a battery of tests."  Counter to this stereotype, my attending was very conscientious of both current literature and a patient's past history.  He was very aware of pretest probabilities and applied this knowledge so that a minimum number of tests was ordered on any one patient.  I remember the classic example of a young female with recurrent SOB.  This was nothing new, and she had already had multiple chest CT's in the past, including the most recent ER visit.  Rather than ordering a routine CT and sending her out the door, my attending was adamant about minimizing her exposure to radiation and admitting/referring her to a specialist to help her breathing troubles.  Having said that, he also noted a recent conversation he had with a colleague who said, "I will run the cost of healthcare down to the ground and order every test imaginable in order to avoid another deposition again."

**Major Theme: Spending time taking care of patients, patients’ education, and understanding**

**Sub-category: Spending time to talk and answer patients’/families’ needs for infromation and support**

Bravo: Last night I had a shift at XXX and worked with an attending whose patience and bedside manner exuded professionalism.  As per usual, the ED was hopping with multiple traumas, intubations, ruptured diverticuli, and febrile neutropenia, just to name a few.  So needless to say, when I presented my patient with the vague and convoluted complaint of nausea, I was certain that this non-urgent patient would be sidestepped or deflected to a resident.  This was not at all the case.  The attending listened intently to my presentation and immediately followed me to see the patient.  He sat at her side while she slowly expressed her concerns and did not leave until he was able to elucidate what exactly we could do to help her.  Where many would have interrupted or become frustrated or short with her roundabout manner of speech, he treated her with respect and gentleness, not rushed or chaotic.  While the workup and treatment plan were minimal, the patient felt comfortable with her care and the plan to be discharged because she knew her concerns were heard and addressed.

Good Example: I worked with a physician who had a remarkable way with his patients.  The one particular example was with a young girl that had just been discharged from Harding after a suicide attempt that morning, and was having some nausea and vomiting.  The particular doctor first asked questions about the nausea and vomiting to try to get to the bottom of it.  After coming up with a treatment plan, he spent a good deal of time talking with the patient and her parents about her suicide attempt and really made a connection with her.  I left the room thinking that the doctor had really gotten through to her and that he had given her some valuable information to think about.  Both she and her parents seemed to be extremely impressed with the physician's conduct.

Good Display of Professionalism: Just today we had a young girl come to the ED with abdominal pain very early in the morning.  She ended up staying in the ED for almost 8 hours before we could figure out what was wrong with her and it ended up with her eventually going to the OR.  I was impressed with the way my attending handled not only her, but her very anxious family.  We had to order a lot of labs and imaging on her and during every step of the process, our attending and I would go in to explain what was going on and to make sure they were comfortable with the plan of care.  Our attending also never got frustrated when this girl's abdominal pain persisted despite large amounts of pain medication; he simply kept looking for ways to keep her comfortable instead of just passing it off.

Lack of Compassion: Unfortunately, I have a bad example of what not to do in our future profession. An attending and I went to see a patient at XXX coming in for knee pain. This patient had a history of drug abuse. When the physician started interviewing her, she began crying about being diagnosed with cancer. Without addressing that emotion, the attending went on to explain that he didn't care about her previous diagnosis and all he cared about was why she was in the ED. He eventually left after she wouldn't stop crying. He told me that she was probably drug-seeking. To make matters worse, he saw in Eresults that she had misunderstood, and she was never diagnosed with cancer. As I was making my rounds later, I found out that he had never come back to tell her she did not have cancer. When I told her, her face lit up and she was ecstatic. Common courtesy and compassion would have gone a long way if they were used in this situation.

Good Example of Professionalism: While working at XXX's I have worked with one attending who I am always impressed at by his professional and caring treatment of patients and families. He has an amazing ability to step back and try to think from the parents and kids perspectives in situations where many people would get frustrated. The other night I was working the 11-7 and at 3 in the morning we have a 8 month old level 5 with congestion for 2 days. The child had no fevers and mom didn't think she was having any real trouble breathing or any other symptoms. On exam the child looked perfectly happy and interactive. Rather than being frustrated, as I have experienced many physicians be when patients come in the middle of the night with very unconcerning presentations, this physician just responded with an understanding "it's hard being a first time parent - she must have been really worried about her kid to bring her in" and spent several minutes talking to the clearly anxious mom and to make sure we had answered all of her concerns.

Poor Professionalism, May Even Consitutute Malpractice: During an evening shift at XXX a patient arrived in the Fast Track department around 11 o'clock via cab from a local OSH. The middle aged male pt had spent the entire day being worked up for a complaint of pleuritic chest pain at OSH. However, according to the pt, at no point did any of the doctors explain to him the nature or reason for any of the tests they were performing, i.e. blood draws.  At some point in the early evening the nurse at OSH came to place and IV and told him he was being sent for a CT scan.  This was likely for a r/o CT PE study; however, at no point was this explained to the pt.  The pt demanded to know what the IV and CT scan were for, and presumably become argumentative with the doctor.  Eventually, without performing the study, the OSH staff sent the pt over in a cab to obtain the CT at XX.  When the XXX physician and nurses looked over the paperwork sent from OSH, they unanimously had the very strong suspicion that the pt did not have the study done because he was uninsured.  This is obviously wrong for several reasons.  First of all, it was unprofessional to not respect the pts autonomy and not explain the reasons for the tests that were being ordered.  Even worse, it was clearly documented that there was suspicion for PE, but the doctor’s d/c him to XXX without doing the study themselves. If this was truly because the pt was uninsured this was no doubt unethical, and actually an incident report was filed with the appropriate medico-legal committee. In the end, once arriving at XX, even though it was late and nearing closing time for Fast Track, the staff was very empathetic to this pts situation. The XXX ED physician displayed excellent professionalism and spent considerable time speaking with and explaining everything to the pt regarding the workup, reassuring the pt that they would talk care of him and provide the diagnostic testing that OSH did not.

Professionalism Slip: I think it has been a recurring theme in many postings this month that some attendings observe great professionalism qualities in the majority of time but sometimes slip under busy/high-stress moments. A few days ago I was working a shift at XXX. The attending I worked with was very nice and friendly. He listened to patients quite well and kept them involved with their plan of care. He also provided me with some great bedside teaching. We got busy in one stretch (spent about 25 min doing an LP, EMS rolled in an unstable patient as we finished our LP, just found out that an accu-check of a previously non-diabetic patient here with an abscess/cellulitis came back at 428, and the nurses handed the attending four ECG's to sign off). Once the nurse handed the attending the fourth ECG to sign off, he told her "I apologize if I am being snappy but this is the fourth one I had to sign off in 2 minutes". Following this, before the attending went to see the unstable patient, I followed him as he went to tell our abscess/cellulitis patient that he now has diabetes. This patient was a previously healthy 29 YO male. The attending said to him "bad news sir, you have diabetes and you need to be admitted to the hospital today". The patient was shocked by the news and seemed not to believe what he had just heard. The attending continued "It's better to find out now than find out after a coma". This whole interaction took about 1 minute. I think it would have been better to see the unstable patient first and then come to this patient and inform him of his new diabetes diagnosis. I eventually came back to this patient and spent a good amount of time talking to him about his new diagnosis.

Misuse of the ER: We had a patient present to the ER simply for medication refill. She was in no acute distress but rather has an expected level of back pain and a higher than normal BP since she had not taken her Percocet or any of her 3 BP meds all day. She ran out of the medications because she could not secure transportation to her PCP’s office last week. My attending was clearly irritated at this blatant misuse of the ER, however he listened patiently to the patient’s complaints to ensure that he was able to rule out life-threatening conditions. On further questioning, the patient sheepishly admitted to smoking 2-3 pack of cigarettes per week. Although my attending was even more irritated at the situation, he did not let his emotions show to the patient at all. Instead, he used the opportunity to educate her calmly on 1) how the money she spends on cigarettes could be used to pay for her transport to and from her PCP’s office and 2) how quitting smoking would help lower her BP.   When speaking with him after we left the room, I learned that he find this approach most effective for a number of reasons. In the ER, there is not enough time to learn everything about the patient. Even though this patient misused the system and took time away from other patients with emergent issues, there might be a variety of extenuating circumstances in her life. He wrote prescriptions for only 2-3 days worth of medications so that this patient would have to visit her PCP in the near future.Staying calm and using the opportunity to educate the patient seems like it might be the best way to handle these situations from a preventive standpoint.

Degrees of Medical Knowledge: It's interesting to me to see how many people come into the ER for eval of what I'd consider very benign, simple problems. It's tempting to get frustrated with them and mentally accuse them of abusing the ER system. However, while there are certainly lots of pain-seekers and system-abusers out there, there are also an awful lot of people who just have no foundation of medical knowledge at all.  For example, I had a patient come in last night complaining of a blister.  That was it - just a ruptured blister. He'd gotten it several days before and had subsequently noticed that the area of white dead skin had enlarged somewhat.  He managed to convince himself that it was infected, and for this reason came to the ED at something like 2 in the morning for eval.  I admit to feeling some exasperation, but then realized that he honestly didn't know the progression of blisters or what an infection looked like.  So - I provided reassurance and education, as did my resident, and the patient headed out happy and with no other problems. It's easy to take all our medical knowledge for granted, but that incident reminded me that there is a lot of simple ignorance out there that contributes to the overuse of the ED system. Guess we can just keep chipping away at it one patient at a time!

Multiple Complaints: I had a 39 year-old female patient at XXX with the following chief complaints: chest pain x 6 weeks, dizziness x 2 weeks, left foot numbness x 6-12 months, increased somnolence, breast lump.  I admit that when I first saw these complaints on the computer, I wasn't very excited about seeing this patient. I had a feeling these multiple complaints would be rather vague and that we would not be able to help her much in the emergency setting. I did the best job I could to address each of her complaints. Her shooting tender chest pain was likely chostochondritis and we prescribed NSAIDS after the appropriate work-up. Her dizziness always occurred when she was getting up (from bed, from doing laundry), and I assured her this was not anything to be concerned about. For her whole foot numbness, we checked her electrolytes. She had multiple symptoms suggestive of major depression, which possibly explained her somnolence. For all of these complaints, the PCRM set her up with a PCP who took Caresource. She also got a mammogram scheduled and an appointment with the breast clinic. Finally, while doing the breast exam, my attending noticed some skin infection and we gave her antibiotics.   All in all, this encounter went better than I expected and I thought we did pretty well for her.  Then my attending asked her what it was that finally made her go to the emergency room. Her eyes welled up with tears and she said she was afraid all these things were caused by cancer. She has an extensive family history of cancer and her sister was just diagnosed. This made me feel like such a dope. I completely missed what her real chief complaint was. My attending did a great job assuring her without saying that she definitely does not have cancer. I also felt my attending did an excellent job by taking her complaints seriously even though their chronicity would have made them more suited to an outpatient PCP visit. He showed great skill in finding out what the real issue was and great empathy for the fear she was feeling.

Taking the Time to Explain Things to Frustrated Patients: We had a patient with severe back pain and 4 days of bloody drainage from the incision-site of her lumbar laminectomy (performed 2 weeks prior).  She had been very uncomfortable with the pain since the operation and had been reassured by her surgeon that it was normal post-op pain.  When the drainage began, she called her surgeon and was instructed to go to the ER in her hometown.  There she was reassured that it was not worrisome, and after speaking with surgeon was told to come to our ER (at the hospital where the surgery was performed) if the drainage persisted for another 48 hours.  On examination here, the wound was clean and healing very well with only mild drainage that was evident to only be blood. The patient could not understand why she was getting what seemed to be conflicting messages: If this was nothing to worry about, why was she told to come all the way to our ER and why did it continue to drain?  She felt that she was either getting dismissed by both ERs or was being misled by her surgeon. My attending took this opportunity to explain to her how the healing process could cause such drainage.  She then explained that it would take a medical professional to examine her to ensure that there was no spinal infection in such a situation, and she also explained the gravity of such an infection.  The patient was very much appeased by this and calmed down considerably from her previously combative state.  The attending also went on to contact the spinal surgeon to determine whether there were any other concerns to address and to arrange a follow up appointment for the following day. (Sorry for the novel - it's kinda slow right now and I'm getting bored...)

Well Managed Code: Earlier today, we ran a code in the ER. I was impressed with how smoothly everything ran, but I was particularly impressed by the way the patient's family member was treated. She chose to stay in the room through the gamete of interventions that were performed. The nursing staff did a great job taking time out to console her and our attending physician also periodically updated her on progress that was being made.  I haven't been a part of great number of codes but this one was unique for me in that the patient's family member was present and in close proximity through the whole affair. I thought it was really great that she was given the choice between leaving and staying and that she was given considerable attention inspite of how busy everyone was. It seemed to me that she appreciated everyone efforts and empathy.

The Human Side of Medicine: I have found it all too common on many of my rotations that the furor and pace of patient care creates a production line of care.  That is why I was pleasantly surprised to have one of my attendings show an example of how to create personalized care in a hectic environment.  Our middle aged female had the classic symptoms of nephrolithiasis, and noted she had experienced them before and felt her presentation was similar, but worse.  He sat greeted her, sat down, went through the exam, and then stopped, explained his findings.  He then told her how a CT scan might confirm the diagnosis, but noted the radiation and cost, as well as the possibility it might not find everything, and gave her the choice of how to proceed.  I thought about how empowering it is to have a physician as a trusted advisor rather than being another part on the mass production line, and decided I want my patients to feel human and empowered.  This encounter took only a minute or two longer, but the feeling when leaving the room was profoundly different.

Futile Care: Tis the season for gastroenteritis at XXX's.  8/10 complaints have been vomiting/diarrhea/dehydration for the past few weeks.  Some cases are clearly worse than others, most are totally benign.  It becomes very easy for the attendings to "eyeball" the kid after my presentation and send them home with instructions of "pushing fluids" and Tylenol for fevers, etc.  However, there is one attending who takes the time, despite the demands of the more acute children, to explain to parents (who are obviously concerned or they wouldn't have waited 5 hrs in the waiting room) the length of the illness, what to expect, symptom management, signs of severe dehydration and when it is appropriate to return for further evaluation.  It only takes a minute extra but is a great display of professional behavior and excellence as a physician

Positive Behavior: During my second shift, I worked with a physician who exemplified professionalism in all aspects of his patient care and team work with ancillary staff. One particular behavior struck me the most. I had seen a patient who had a history of DVTs and was diagnosed with a new, extensive DVT on Doppler that morning despite a therapeutic Coumadin level. The patient needed to be admitted for an IVC filter. The patient, however, was quite frustrated with this. He did not understand why it was necessary as he was asymptomatic and he did not understand why his doctor did not tell him about this earlier so he could have made arrangements in his life prior to being admitted. I told the physician of the patient's frustration. Rather than just being paternalistic toward the patient and saying he must be admitted, the physician took at least 10 minutes to talk things through with the patient. He explained the danger of his new DVT, why the patient's PCP did not know of it beforehand and why the hospitalization was necessary. He took the time to ask the patient about his apprehensions as well as suggested that he talk with his family prior to coming to a decision. Taking the extra time allowed the patient to understand everything better. Even though the ER is quite busy, taking the extra time with patients can make a tremendous difference.

Professionalism – Patient Autonomy: Yesterday I worked with a physician with a true regard for professionalism.  He spent quite a bit of time telling the patient what he thought was going on, what he thought could be ruled out, and then discussing their testing and treatment options.  He wanted to come to an agreement on the management plan and strayed from the paternalistic manner some physicians deal with patients in.  He did this each and every time we went in together to see a patient even if they were coming in for simple nonemergent outpatient complaints.  It was refreshing to see this type of commitment to bedside manner and patient autonomy on this rotation.

Procedure Professionalism at XXX: I have noticed that at XXX in the ED, there are patient service employees who work with parents to keep them oriented, and aware of what is happening with their child.  When procedures are to be performed, they are there to answer questions, prepare parents for the procedure and offer emotional support.   I had a three year old patient with a laceration on his forehead which needed sutures (yes it was a coffee table incident).  I really felt that the patient service worker was an asset.  She was present before and after the procedure to help the parents cope with the possibility that we might have to hold down their son while he screamed... As it turned out, the little tike tolerated his sutures very well and did not require holding down.  I was very impressed with XXXXHospitals professionalism in dealing with the difficulties that come with providing care for sick kids.

Professionalism – XXXHospital: During my rotation at XXXHospital I have worked with different attendings each shift. One particular shift I worked with a physician who had the best bedside manner I have witnessed in medical school. Each patient he saw in the ED whether they had a migraine or stroke, rash or a broken bone he was polite, patient, and respectful. He would make sure that no patient left the ED without fully understanding the diagnosis and the treatment of the illness. Although he spent more time with each patient than the average ED physician, he was very efficient and self sufficient! I was impressed at his level of dedication, willingness and energy he provided to each patient. Although we all strive to be like this, sad to say, most physicians I have encountered are not! treatment of different patients differently or "just don't care" attitudes at the end of the day or shift seem to predominate.

Examples of Professionalism: I had my medic ride along today, and saw several examples of outstanding professionalism.  There were two calls where the medics either had nothing to do for the patient, or only provided transport.  In both cases the medics were patient, polite and made some small gestures to help the patient or their family.  I've seen this is in the ED too, when staff take a little extra time to explain a test result or offer advice to a patient without acute illness.  My psych attending said that every patient interaction should be therapeutic, and I think that can be possible, even in the ED, and even when patients aren't acutely ill.

Qualities of Professionalism: This month, I have worked with an attending who embodies many qualities of professionalism.  During each patient encounter, she makes the most of her time; she sits down near the bedside and listens to the patient carefully while maintaining good eye contact.  She speaks to the patient in clear, understandable language regarding any procedures, test results, and diagnosis.  She makes sure to address the patient's concerns.  There have been instances when a patient becomes angry for waiting for a long time – She begins by calmly apologizing for the long wait and explains that there were more emergent situations that she had to attend to first.

End of Life Discussions: I witnessed an example of professionalism last week regarding the end of life discussion. The patient had recently received chemo for lung cancer and came in with septic shock due to pneumonia. The physician explained the need for an elective intubation with the patient and his wife in detail and agreed to wait until the patient's son arrived before starting. Later in the shift, the patient's son and wife approached the doctor at his dictating station to ask him some questions. The doctor stepped away from his computer and, despite being incredibly busy, took the time to answer all of the family's questions without rushing the discussion. I was relieved to see the doctor step away from his other work for a moment to take the time to have a real discussion.

How Much Time: I have noticed big differences in how much time doctors spend with patients in the ED.  Some doctors seem to be in and out of the room in a minute or two, and don't answer questions unless asked.  Other doctors anticipate questions, and give detailed explanations of their thought processes.  I don't think there is a proper amount of time to spend with each patient, but appearing to be hurried can lead to problems.  One patient I saw began coughing and then vomited as he became anxious during a doctor's rushed questions.  He hadn't complained of any vomiting up to that point, and the episode may have sent the doctor down the wrong track in diagnosis.  A doctor may think that a patient's complaint amounts to little, but a calm and reassuring explanation often alleviates the patient's worry.

Giving Bad News: A couple of days ago, a 68 year old female with recent femur fracture surgery 1 week ago, presented to the ED with extreme back and abdominal pain.  After the ABCs, we did a quick FAST scan which was not conclusive.  We then sent her to get an abdominal/chest CT. While at the scanner, she coded and unfortunately passed away. During much of this time, I stood with the twin sister and son of the patient as the ED staff put lines, monitors, etc.  I was explaining to them each of the steps and why she was going to the CT scanner.  After the whole team rushed to the scanner, the patient stayed in the trauma bay.  It was realized later that the patient did not have a ruptured AAA, but instead a huge PE.  She was not on any anti coagulation post op from her ortho surgery, although she was pretty much immobilized.  A few minutes after the patients passed, myself, the ED attending, and the chaplain went to speak to the family-very very very sad/difficult experience.  I was tremendously impressed with both the ED attending and the chaplain. They sat with the patient, comforted them, discussed all the news/events, explained what happened.  The ED attending spent about 20 minutes talking with the family even though he had lot of other patients to see and things to do.  He had a few opportunities within the discussion to excuse himself and leave further discussions with the chaplain, but he chose to stay until all they're present questions were answered.  He showed tremendous empathy and professionalism throughout the discussion. I hope, when encountered with a similar situation, to emulate that attending.

Bad News: Death often times can bring out the best or worst in a physician. While working a shift at XXX a young man was brought in by squad after crashing his car and suffering a traumatic brain injury.  He was supposed to be XXX Flighted to XXX, but he lost a pulse while still at the scene and so was brought to XXX to be pronounced dead after futile CPR was performed for a short time.  So the attending I was working with was left to tell the family about the patient’s death.  Even though XXX does not have the busiest ED, the doctor still had many other things to do, but he took the time to talk to the family and console them to the best of his ability.  I am sure he could have left it to someone else to tell the family, but he took it upon himself to tell the family

Continued Patient Assessment: One of our goals this month has been to accomplish continued patient assessment while seeing new patients, even during relatively hectic times in the Emergency Department. I have also seen many of my attendings place emphasis on continued patient communication, as well. We have all seen patients get frustrated at potentially long waiting times in the ED waiting areas. However, what can be equally frustrating for a patient is sitting in his or her room for a long period of time while waiting for pertinent labs to return. It is not only important to continually assess every patient's vital signs and clinical picture, but also his or her state of mind. Oftentimes, lab values take varying amounts of time to return (as do radiology reads), and I feel it is important to update patients on values and reads as they return, not all at once when their final disposition has been determined.

Lack of Completeness: We had a pt who was a middle age woman who presented with chest pain.  Her story was fitting pericarditis w/ the cold symptoms a couple weeks back, etc.  EKG fit also.  I explained my reasoning to the attending and he was like fine.  Of course, we did the normal ED thing of rule out MI, STROKE, PE w/ labs etc which is fine by me.  But in the end, we didn't do anything for the lady.  I even inquired w/ the doc as he was writing the discharge papers about the diagnosis of pericarditis and anti-inflammatory meds.  He brushed me off and the pt left w/o any idea of what was causing her pain.  I feel like this was a lost opportunity to help someone w/ chest pain and educate her.  I feel this lack of completeness is what leads young people to ignore chest pain until it’s too late.

**Sub-category: Spending time with patients, listening respectfully, learning their history and concerns**

Poor example: I had my first shift yesterday evening.  To begin the shift, the attending had me shadow him for a couple of patient interactions.  For both patient interactions, the attending neither introduced himself or myself to the patient.  Each time, he went in, asked a few questions regarding the HOPI and quickly began his physical exam.  He did an excellent job of bedside teaching to me by explaining his differential and what he was examing for.  However, there was no other interaction with the patient.  I could see the patient was anxious regarding his condition.  Being the first time to work with (let alone meet) this physician, I did not feel comfortable at all to say/day anything about this.    I thought this was noteworthy in light of our recent lectures yesterday morning regarding "the approach to the undifferentiated patient

Crying Wolf: I've noticed there have been numerous patients with visits >10 per year ranging from chest pain to abdominal pain. It seems that most doctors usually get a bad taste in their mouth when they are able to recognize a patients name on the chart. However I've noticed one particular doctor who took the time to go over the entire history and physical as if he had no prior knowledge of undifferentiated chest pain and abdominal pain. It was nice to see this commitment to have no bias or cynicism.

Patient Compassion: During my first day of this elective rotation, I witnessed an exemplary display of compassion for a patient. A woman presented to the emergency department because she had been having heavy vaginal bleeding since she had a spontaneous abortion at five weeks. She had been taking fertility drugs for a long time and this was her first pregnancy. She indicated that it had likely been her last chance to have a child, since she was 35 years old and did not want to take the risks associated with pregnancy after this age. The patient was very upset. The third year resident on her case was very supportive and kind to the woman, making sure to show her how sorry she was for her loss. I thought it was very professional to take the time to make sure that the patient had the opportunity to voice all of her concerns. The resident fully addressed all of the patient's social issues. She showed a compassion in her patient care that I felt was worthy of imitation.

Just Listening: I am at XXX, we do 10 hour shifts, and after a couple of shifts I realized that I better start bringing a snack in my pocket because there is no time to go spend my meal tickets.  They are very busy most of the time.  That being said, I was impressed with an attending one day when we went to the patient's room to discuss some findings.  The patient had been struggling with stage IV prostate cancer for the last 2 years and was starting to go downhill.  The patient was gone to CT when we went to his room, but his wife was there.  From her expression, you could see that she was very down feeling that this could be her husband's last run.  It wasn't much, but the attending simply and sincerely asked, "Are doing okay?"  It took less than 5 minutes, but she let out a little bit of her concerns, frustrations, etc to us.  With a "we are going to take care of your husband" from the ER doc, she said, "Thanks for taking the time to talk with me."  It wasn't "care" for the patient, but it meant a lot to her definitely took care of the "whole" patient.  It meant a lot to see that and I hope I can do the same and take small moments to comfort.

Autonomy: I worked with this attending during my most recent shift. Sometimes I felt like I was on an Internal medicine rotation - in terms of the depth that he would go into in eliciting the H&P. He never made the patient feel rushed - would always note down every detail, and then would constantly pop into the room to update the patient on the status of the CT scan, lab work etc. At the end, he would involve the patient in decision making, and give them a sense of autonomy. Yes, he did take quite a bit longer than the other attendings, but I felt like the patients were happier upon disposition.

Listening to Parents: One of the most important parts of working in a XXX ED is listening closely to the story the parents tell.  If parents are concerned, it is important to take this into consideration when developing a plan.  A parent knows their child best and if they truly believe that their child is "different," it's important not to disregard this impression even though a child is seemingly "non-sick" in a professional's eyes (i.e. consoles, eats, drinks, etc).  I have seen several ED attendings go above and beyond to really listen to parents in this ED even though the child appeared well or "non-sick" to us.  It has been a valuable lesson in taking pediatric history-taking!  And professionalism!

Chronic Pain: I was impressed with one of the ED physician’s professional approach to patients with chronic pain.  Even though the chronic patients presented with the same complaints as their numerous previous ED visits, the physician still took enough time to gather a pointed history and perform a thorough focused exam to ensure that these patients had no new acute complaints that could be addressed in the ED.  All this while the physician would chat with the patients with ease and approached these patients without a change in demeanor.  I was impressed with how the physician was able to prevent himself from showing any hints of being jaded after all these years and treat the person in the chronic pain patients versus just the chronic pain seeking behavior in these patients

Kidney Stones: We had a patient who came in with severe right flank pain radiating into his groin.  He was a known IV drug abuser, and had come in before with various complaints that always seemed to get better with narcotics.  The attending that I was working with though, was very professional about the whole thing.  He made it a point that even IV drug abusers get kidney stones sometimes and that we had to take his pain seriously at least until the workup was done.  In the end, he did have a kidney stone, a pretty big one, that we saw on the CT scan.  He ended up passing it before he left the ER, and never asked to go home with any narcotics or anything like that. I just felt that it would have been really easy to blow him off as a drug seeker and not take his pain seriously.  I think it's important to remember that even drug seekers occasionally get sick:)

Widening Our Differential Diagnosis: One thing I have noticed in the ED is that when you have several patients to see and evaluate, one can easily feel the pressure to quickly diagnose a patient's problems.  One example I saw occurred earlier today.  A 20 y/o WF with no pmhx presents with epigastric pain, nausea and vomiting for the past 12 hours. The patient had been doing well up until the previous night, where after having a greasy dinner at a steakhouse, immediately had one episode of vomiting (non-bloody) followed with epigastric pain.  The patient was able to sleep, and awoke the next morning to eat a breakfast consisting of leftovers from the dinner the previous night. This was again immediately followed by an episode of vomiting, nausea and abdominal pain.  On admission, the patient appeared to be resting comfortably in bed.  Further history elicited similar symptoms for the past 6 years along with a worsening of the symptoms when she had been pregnant the previous year.  Her symptoms had always been relieved with Tums, Rolaids and/or Mylanta.  On exam, the patient's vital signs were stable, and no pathology was noted on exam, except a mild tenderness in the epigastric region with a negative Murphy's sign.  The patient was advised to start taking Prilosec OTC for presumptive GERD and to return if her abdominal pain worsened with associated increase in vomiting and/or nausea.  Nine hours later she returned visibly upset with worsening abdominal pain, nausea and vomiting despite eating only chicken broth since leaving the ED.  An H&P was again taken from the patient, and it was discovered that she had actually had a CT of the abdomen last August for similar symptoms.  On review of this CT, it was found that the patient had gallstones even at that time.  The patient was admitted to the hospital for acute cholecystitis, with a consult for surgery pending for the next day.   On review, the patient's symptoms initially appeared to be similar to GERD, and her history pointed to a long-standing problem with reflux.  However, had we spent several more minutes with her when she first presented to the ED, and asked more directed questions about what procedures had been done, we would have moved cholecystitis/cholelithiasis to the top of her possible diagnoses. This case is indicative of multiple aspects of professionalism, including competence, integrity, and respect

**Sub-category: Taking full responsibility for patient care and informing health care providers and caregivers**

Professionalism Under Fire: took part in a rather challenging case today and I thought my attending provided a good example of how to conduct one's self professionally in a hostile situation. The pt in question was a known drug seeker with a reputation for hospital hopping. However this patient additionally had a genuine cause for chronic pain consisting of pseudotumor cerebri. The pt presented today after having consumed her last dose of oxycodone. She was on a pain contract in which she was given a limited amount of pain medications and was not to have them refilled until later this month. So my attending was faced with the dilemma of a pt who had abused her pain medication, but at the same time was truly suffering from her medical condition. Although the patient was hostile to my attending, he kept a polite tone with the patient and presented options to the pt, other than just giving her pain medications. He obtained a neurological consult and medicine consult to evaluate her in an acute manner in the ED. While the evaluation was taking place he temporarily treated her pain. While in the ED he set up proper follow-up for the patients in 2 days time and discharged her with enough pain medications to carry her until her appointment. I felt a few laws of professionalism were followed well here. There was a sense of justice, in that the doctor did was he thought was right and best for the patient by not indulging her addiction. By not indulging her addiction he prevented future harm by discouraging the habit of doctor hopping. He helped the patient through the acute episode by providing only the medication necessary to get her to proper follow up. Lastly, he was respectful to the patient despite the fact that she had lied to him and several other staff members in an attempt to abuse the system, in addition to her open hostility towards him.

Primary Care in The ED: I have been impressed with the method in which a physician I have worked with approaches those patients that approach the E.D. for primary care needs because they lack established linkage to primary care due to lack of insurance or other reasons.  In particular, there was a patient that had multiple E.D. visits secondary to chronic back pain.  Previous imaging demonstrated severe disk herniation and convincing evidence for a source of this patients pain.  While my experience to date with patients presenting with similar complaints entailed discharge with a couple of day supply of pain meds and a list of neighborhood primary care physicians, this physician approached the problem slightly differently, and in my opinion, more effectively.  He took the time to sit down with the patient, address his complaint, and explain why using the E.D. for primary care was ineffective, because in the E.D. setting, the physician could not provide access to alternative therapies to narcotics (such as physical therapy).  The physician then, in addition to solely referring the patient to the social worker for linkage to a PCP, provided a list of local clinics that provided care on a sliding scale basis and emphasized the importance of finding a PCP for management of this chronic condition. While I was impressed with this physician's compassionate approach to a problem that many would write off as non-emergent and thus unimportant, what struck me most was this physician's service to his profession.  In the current environment in which providing good outcomes in a cost-effective setting has become a secondary concern as physicians are constantly pressed for time, this individual took the additional 5 minutes to personally redirect this patient to an alternative source of care, which was not only best for the patient, but best for medicine as a whole.

A Professional Approach to Pain Control in the ED: Those two words can make emergency physicians cringe. As I prepare to embark on my career as an emergency physician, I recognize that I will be often faced with the issue of pain management in patients coming to the ED complaining of pain. It can be hard to approach patients who we feel are just seeking pain meds professionally and objectively. In fact, I have seen many attendings rely only on their opinion of the patient’s pain, and be very dogmatic towards patients. I believe that doing “no harm” has two parts. First, it requires treating what is there, i.e., pain, and second, not treating what is not there. This can be a very difficult balance to find. During my shift last night at XXX, I had a patient complaining of leg pain. He automatically raised a few red flags: out of town, out of pain medication because he had missed his doctor’s appointment two days ago for unclear reasons, wearing sunglasses in the room. He had an obvious mechanism for pain, but his story was a little suspicious. I found myself wanting to believe this patient, and while still felt some hesitation, decided I felt comfortable with giving him a few pills of his pain medicine. The attending was very wise and professional in his approach. He suggested I try to reach the patient’s doctor (the patient gave me his name and number), and try to check with him. I was able to reach the ortho resident at the hospital at which the doctor had privileges. The resident was able to look up the patient’s records, determine how long the pain pills should have lasted the patient. Together, we were able to determine a plan of approach, giving the patient a few pills, and have him call the clinic to follow up. The attending was then comfortable and informed, the patient’s doctor was also informed, and the patient was very satisfied with the results. I believe this is an excellent model of a professional approach to treating pain which I will use in the future.

Diligent Forward Thinking: One of the ER docs I worked with saw a patient with mental status changes and an abnormal neurologic exam worrisome for a stroke.  In addition to doing all of the appropriate work-up and immediate ER care necessary, this physician took the time to call the patient's PCP and neurologist, to talk to the radiologist before the CT was done to tell them what he was looking for, and to get input from the admitting neurology team to get all tests ordered and consults placed in a timely manner in the ED instead of unnecessarily waiting until admission for them to be ordered.  I was quite impressed with this continuity of care, keeping other docs in the loop, and how this sped up the care so that this patient could get the best and timeliest interventions

Patient Advocate: One afternoon when I was in the ER, a patient who had 4 previous miscarriages presented during her first trimester with bleeding and cramping.  The ER physician that I was working with felt that it was very unprofessional for her OB to send her to the ER.  The most he could do was offer her an ultrasound which would be a further expense for her.  He called her OB and explained that there was nothing he could offer to stop the miscarriage and that she should have been seen in his office for counseling and support when she called.  Her OB apologized and the patient was transferred over to his office for an urgent appointment.

**Major Theme: Going above and beyond, caring, and altruism**

Are Certain Topics Off Limits in the ED?: When is the appropriate time to tell a pediatric patient's parents that you're concerned their child might have a development disorder?Little Billy presented to the XXX's ED a few nights ago: a 15 month old boy with a pretty open-and-shut case of simple febrile seizure. My associated findings, however, led to an interesting dilemma.Billy's mother and father told me that he doesn't feel pain. Billy had accidentally cut himself several times but never fussed about it--they realized he had cut his hand only after they noticed blood smeared across the kitchen tiles. He once grabbed a hot curling iron--wrapping his fingers right around the hot elements--and that barely phased him. Later, during my exam, LB's parents mentioned that he's been having some hearing problems. They said LB doesn't seem to hear them call his name. This history raises alarm bells for autism spectrum. His parents didn't mention autism though. They also said they hadn't mentioned these issues to their regular pediatrician. So what to do?I wanted to discuss this potential diagnosis with the family. Early diagnosis can be very beneficial to an autistic child and their parents. The earlier intervention begins, the sooner the child is set up with the appropriate resources, the more they can benefit from those resources. And in this case, though the child had a regular pediatrician at an affiliated clinic, I read through the clinic notes and saw no mention of abnormal developmental issues or concerns.The physician with whom I discussed the case felt otherwise. He pointed out that "this is not an emergency room diagnosis." I trust he meant that we weren't capable nor had the time or expertise to make such a diagnosis in ED. And that's true, but we could have recommended the family follow up with their regular pediatrician to address these concerns. But maybe I was wrong; maybe the child was perfectly normal and not at all on the autism spectrum. Discussing it in the ED, when they presented for new-onset seizure, might have created needless worry exacerbated more immediate and justified concerns. So I'm not sure what was the best course of action. On the one hand, the Emergency Department isn't an ideal environment to address pervasive developmental disorders. On the other hand, early intervention is important for autistic children and this was an opportunity to at least start that diagnostic process.In the end, we did not discuss with the parents the possibility of autism. We simply informed them about simple febrile seizures and recommended they see their regular pediatrician in the next couple days for follow up. But was that the appropriate course of action?

Professionalism in the ED: While on a shift at the XXX ED, I picked up a patient who had been noncompliant with his insulin because he was unable to afford the one he had been perscribed which cost over $200/month.  The physician that I worked with then spent the next 30 minutes on the phone with the pharmacy to find the most affordable insulin with which we can send him home.  Many times the ED physicians are so busy and over worked that there is not time to straighten out the loose ends or misinformation from the patient.  It was nice to see a very busy ED doctor take the time to try and improve the healthcare of this patient.

Positive Professionalism: During a busy ED shift, a patient was supposed to get admitted to the hospitalist service for further management of her SOB from fluid overload after going four days without dialysis.  However, the patient and her family were convinced she did not require hospitalization.  Against the advice of the ED physicians and the hospitalist team she decided to leave against medical advice.  However, rather then letting her storm out of the hospital, one of the ED doctors took the time to complete discharge instructions (which he was not obliged to do), fill prescriptions, and walked her through what she could expect if she acutely decompensated   It was refreshing to see this physician work with another service to take responsibility for her care despite the fact she was being a difficult patient.  He later emphasized that although physicians may not be liable for the outcome after a patient leaves AMA, that it was still important for doctors to provide these patients with proper instructions and medications before they left.

Professionalism: I have enjoyed my interactions with the residents and attendings on this service.  I think working here in the ED has helped improve my opinion of the physicians in the ED which unfortunately had a negative slant due to the heresay on the floors.  The ED physicians are good at their art--focusing on a differential that includes emergent dx and how to rule them out or work them out.  While the majority of their cases are not the stereotypical cases that one sees in Hollywood presentations they approach each patient with (for the most part) with an open mind and allow them to feel that they have a reasonable condition which needs to be addressed--even if this is contrary to their own belief.  One resident in particular that I have had opportunity to work with is a great example of bedside manner and for showing genuine concern for the patient, regardless of their complaint.  In the ER where interactions are usually brief and the physician may never see the patient again this type of approach to the patient I think helps the patient to feel more at ease in the medical setting.  This is important in the ER because a large portion of the population that visits the ER may not have a primary care physician.  Being treated reasonably in the ER may help encourage them to seek out regular medical care with a PCP.

It Was Cold Last Night: I was on a night shift when the air temperature was below zero at a downtown ED. The night wasn't very busy and there were some beds open in the ED. We had two or three patients with very vague, non-emergent complaints and when I tried to get their H and Ps, their stories were not making sense. I presented the patients to my attending and he said they might be homeless and just looking for a place to stay for a few hours because it was so cold outside. I went back to the patients and talked to them some more and realized that my attending was probably right. Although they didn't admit to looking for a place to camp out, they did say they were homeless. Given their complaints and the likelihood that there was nothing medically wrong with them, my attending didn't order any tests and allowed them to stay in the ED for a bit as long as the beds weren't needed. I thought this was a very kind and professional gesture on the part of my attending. I don't think the hospital management would have been to happy to hear that it's resources were being used without any compensation or treatment given to the patient. My attending showed what it is to truly care about people while risking being reprimanded.

Taking Time for Our Patients: My first week of the rotation I had an 82 yo gentleman come in after tripping on his rug and ripping a big chunk of skin off his dorsal hand to the point where we had a great view of his tendons and vasculature.  It appeared as if everything was intact except for the giant flap of skin that was hanging loosely by a small piece and was turning purple.  I know there are some people in our class who would have jumped at the chance to repair this one, but I felt a little unprepared - although willing with some help and supervision.  The skin was so frail and macerated, I wasn't very comfortable with this being my first lac repair of the month.  And my attending didn't want to touch this guy either.   So we're at a rural hospital.  We spent the next 2 hours trying to find a hand surgeon who might be able to do the repair.  The surgeon that we did talk to from XXX told us to "just do it" and he'd reexamine in follow-up in 2 days.  By this time it was the end of my attending's shift.  Rather than pass this one on, he stayed another 2 hours and helped do the repair.  It took us a long time to get it right, but at the end I think it didn't look that bad.  I just thought this was a great example of professionalism for this attending--afterall his shift doesn't end until all his patients are situated.   Unfortunately my patient came in the following week with a seizure (completely unrelated - he had a history of seizures).  The hand repair was looking pretty good and it didn't look like he'd need a skin graft.

Flu: While in the ED last week I had a 9-year old patient brought in by her mother. She was diagnosed with Influenza A and discharged with the appropriate antivirals. The issue for this discussion board arose when I asked the attending if we should offer chemoprophylaxis for the mother as she had not come down with the virus yet (CDC website states efficacy of 68-89% in preventing the flu in those exposed to household members diagnosed with flu). She was the primary caretaker for her child and a few other children at home. The attending said that he does not normally offer prophylaxis for the flu, and we did not offer it in this case. Two days later I was working in the same ED and the mother came in with fevers/chills/body aches, etc. Should we have offered the prophylactic antivirals to the mother???

Taking Time for Humanism: It was the end of my attending's shift and he was basically making sure all his patients were squared away to be either admitted or D/C'd from the ED before the next physician came in. As he went down the list he came to a patient who presented with non-specific complaints of nausea and vomitting and ended up being 10 weeks pregnant. He had previously told her in the evening she was pregnant and set her up for a TAUS. As he went back to tell her she could go home the young new mother was curious if she could have a picture of her ultrasound to take home with her.  The tech had not printed one out and we initially assumed it was impossible to get her this picture. However, my attending realizing that this was the patient's first child and thinking about what he would want if he was in that situation went to additional efforts that I have not seen in the fast paced world of the ER. He contacted radiology and when they weren't any help he decided to print the picture out from pacs on his own. After spending 15 minutes with sizing the image correctly, a paper jam, getting ink all over himself and then fixing the printer he found the perfect U/S image of the fetus. He gave the image to her new mother who was elated at the doctors kindness and extra efforts. I really thought this was a great example of a physician who remembered that even though he was busy and nearing the end of his shift, never forgot that he was treating people and not just patients. This commitment to his responsibilities to all aspects of patient care is a very positive example of professionalism and humanism in medicine.

**Major Theme: Communicating and working in teams**

All Gowned up and nothing to do: While discussing a patient with an ED attending I was alarmed/excited when over the PA system I heard, "Trauma Level 1 10 minutes."  The attending told me to go get gowned up for this exciting and potentially educational event.  While getting gowned-up with gloves, hat, booties etc (which I was told were required for a level 1 trauma) I heard the roar of the helicopter delivering the patient to the hospital.  While waiting for EMS to deliver the patient to us I am informed that our patient has some sort of head bleed and that neurosurgery was present.  Just after the patients entrance into the bay the neurosurgeon says "WHO THE HELL CALLED THIS A LEVEL 1."  The nurse recording info reports that he had in fact given the OK for a level 1 designation in order to fly the patient to the hospital.  After some arguing the patient is presented as simply an elderly women with Alzheimer’s who fell in her nursing home.  She was not in very bad shape trauma wise and it was clear that the neurosurgeon did not in fact want her flown to the ED.  This was the slowest and most relaxed level 1 trauma you will ever see.  I was all gowned-up with nothing to do. This was obviously an instance of miscommunication and inappropriate use of very expensive and limited resources.  The error was only compounded by the neurosurgeons accusatory tone and scramble for a scape goat.  He should have addressed the error in communication and looked for ways he could have improved from his end after effectively stabilizing the patient!  ABC does not stand for Argue Berrate Condescend.

Families and Life-Threat Patients: An adult male patient is brought in by squad from home short of breath. Upon arrival vitals are obtained, and the patient is placed on 100% non-rebreather. The patient's daughter was brought to the bedside and the possible clinical courses were discussed wit both the patient and his daughter. This discussion centered around code status and measures that may have to be taken, given the patient's history of COPD. Intubation and chest compressions were discussed and the patient's code status was established as full code. A short time later the patient's work of breathing increased and he was placed on CPAP. After a short time it became evident that the patient was failing non-invasive measures to establish airway and breathing. The patient was informed that they were going to intubate him and his daughter was asked to step out of the room while the procedure was performed. The patient underwent rapid sequence intubation and was placed on the ventilator. As respiratory therapy was adjusting ventilator settings and the physician was leaving the room the cardiac monitor alarms sounded and the rhythm showed an abnormal ventricular rate and rhythm. The patient was assessed for pulses and was found to have none. Chest compressions were initiated, the attending was called, and the crash cart was brought into the room. ACLS was initiated and after one round the patient was found to still be in PEA. Another round of ACLS was performed. The attending asked that an ABG be drawn, central venous access be established, and that the patient be bagged at a rate of 6 times pr minute. At that time the attending stepped out of the room and began talking with the patient's daughter. After a brief discussion with her, the attending brought her into the room. At the completion of the third round of ACLS, with he patient's daughter in the room, pulses were established.  While the patient was critically stable the crash cart was removed, central venous access was obtained, and ABG was sent, and an A-line was established. The patient's blood pressure was being maintained at approximately 90 systolic, with 2 lines running in wide open. The patient was placed back on the ventilator. While the attending was calling the ICU, the resident left to complete information on other patients. Another resident was obtaining US images with the medical student while the nurse was completing her documentation and organizing the patient's many devices at the bedside. The patient then returned into PEA with no measurable blood pressure on A-line. Chest compressions were initiated, the resident was called, the attending was called, and the crash cart sent for. Upon entering the room, the resident established where in the ACLS protocol the team was and then proceeded to ask the patient’s daughter, who was at the bedside, that she may stay if she like or step out if she would prefer. After one round of ACLS pulses were re-established, and the patient stabilized. The resident asked the daughter if she understood everything that was happening, and told her that the patient would be heading to the ICU, and asked if she had any questions. The patient was taken to the ICU a short time later.

Teamwork: Call me lucky, but I have yet to encounter a prime example of unprofessionalism on my ER month so far.  Sure, frustrations have manifested with the occasional sigh or "noooooo! not this patient again," but all in all, these salutations have been handled with respect, proper management, and compassion.  What has impressed me the most, however, is the way the docs work as a team.  Almost all of them show up to the trauma bay during a code when they're free, they all offer to tie up loose ends for a colleague’s patients at the tail-end of his/her shift, and they take on more patients if one of them is getting slammed.  I guess it just goes to show that a healthy and supportive work environment breeds happy employees.

The Team Approach: I have found working in the ED very interesting in the fact that the system is so different than other parts of the Hospital.  What has impressed me is how close everyone works together.  At least in the hospital I am in; the doctors, PAs, nurses, and everyone else work as a close group.  There is more interactions between everyone and people seem more willing to share responsibilities.  I even saw an attending answering the phone for the unit coordinator because she (the unit coordinator) stepped out for a minute.  The attending could of easily just let the phone ring as she had not paged anyone, but  by helping out, the person on the other line got their question answered much quicker.  To me everyone working together to get the best care for the patient in an open and friendly atmosphere is true professionalism

Interspecialty Communication: Last night I saw a tragic error narrowly avoided.  A patient under treatment for metastatic lung cancer came in with acute severe post-prandial abdominal pain, meeting SIRS criteria with an elevated lactate, looking quite ill, but with a benign abdominal exam.  Our ED resident had a high clinical suspicion for acute mesenteric ischemia, and obtained an abdominal CT angiogram to evaluate for dissection/aneurysm.  The Radiology resident, who was swamped with trauma images, reported that there was no dissection or aneurysm, which is what he thought he should be looking for.  On later discussions, it was learned that he noticed a possible occluded SMA, but dismissed it as artifact caused by poor contrast bolus timing.  The ED resident had continued concerns about the patient, but was having difficulty establishing a diagnosis.  The surgical team was consulted, but the surgery residents were dismissive after their exams revealed no peritoneal signs.  Eventually, the attending suggested RUQ ultrasound with doppler flow imaging of the SMA, which confirmed SMA occlusion and vascular surgery was involved, but diagnosis and treatment were delayed many hours after presentation, considerably worsening the patient's prognosis.  I have trouble finding particular fault with any of the individuals involved, but instead feel that this is but one example of the poor interspecialty communication that is pervasive in medicine.  Had the ED resident given the Radiology resident better clinical data about the patient, he would have seen the SMA thrombus.  Had the Radiology resident informed the ED resident about his finding of "artifact," she would have realized that it was thrombus.  Had the surgery team known more about the resident's specific concerns for mesenteric ischemia or if they had paid more attention to the ED resident's clinical judgment that the patient was critically ill, they may not have dismissed the patient after finding a soft belly.  I feel that poor communication like this is endemic to medicine, that most of us know of and acknowledge this problem, but because of the "unofficial curriculum" that we all learn as students and residents, we end up incorporating those same bad habits, perpetuating the problem.  Sorry, it's late on a night shift, and I've started to ramble.  Those of you who work with me down the road, please call me out if I'm doing a shit job of communicating; you'll be doing me and our patient a favor.

Dealing with a Difficult Patient: At the XXX ED, a female prisoner patient was being very difficult with the nurse treating her. This patient was verbally abusive and could be screaming throughout the ED. She also started becoming abusive toward hospital property, creating a hazard for other patients and staff. I though the XXX ED physicians and staff handled this situation in a professional manner. For one thing, they did not return the negative language. When they saw that one of their colleagues was in a difficult situation, all the staff in the near vicinity came to lend a hand by subduing the patient, calling security, and warning others about food thrown on the floor by the patient so that no one would slip. This was a nice example of a team working together to help one of their colleagues in a difficult situation.

Too few Resources: Yesterday we had one patient come in by ambulance and the other by XXXFlight and they were both ACS patients.  It was after business hours, so the on-call cath team was paged to come in.  But because there were two patients needing the cath lab, the ED unit clerk was scrambling to find a second catheterization team.  A second team was unable to be located.  The ED team had to decide which of the two patient's conditions was more severe and send them to the cath lab.  I felt extremely uncomfortable watching as the doctor told the less-ill MI pt that he would have to wait his turn.  I understand that "time is tissue" and I knew this guy's 90 minute window was diminishing.  I know that this is not common, but it seems like there should be measures in place to deal with such events when they happen.  I wished that there could have been more communication among XXXFlight and other local hospitals to take that guy to a hospital that had available resources.

Teamwork: I have been impressed by the team attitude displayed specifically by the attendings and pa's this month. When one individual has drawn the proverbial short stick and received more complicated patients, has been running behind, would appreciate a second opinion, or has a patient who so obviously would feel more comfortable with a health care provider of a specific gender, others have shared the burden to help ensure that patient care is the first priority. The result of these individuals all helping to share the burden in my opinion has translated into more quality, efficient patient care and a much more pleasing environment in which to work.

**Major Theme: Unclear stories**

To Treat or Not to Treat: I met a patient during a shift who was in her early twenties and in for her 6th or 7th visit within the past 2 months.  She was a drug-seeking patient, but she had a medically acceptable reason to seek relief from her pain: sickle cell disease.  I mention this patient for discussion in order to highlight the frustrations that both medical professionals (med profs) as well as sickle cell and chronic pain patients experience with pain management.  I spoke with a med prof who described treating chronic pain patients as frustrating, given that there is no way to objectify pain in light of the real pathophys of the disease process.  Another med prof I encountered was openly frustrated and mistrusting of the patient, wondering how one could experience so much pain in such a short interval of time.  Of course we understand that tolerance may build with chronic drug use, but we also know that in many situations, the pain our patients feel is real and that tolerant or not, they desire some relief of their pain... We treated the patient as she had been treated in the past, with fluids, o2, pain meds, and eventually another admission.  I'm sure the question lingered: when, where and how do we draw the line between legitimate and illegitimate pain management??

Cost vs. Duty to Act: Scenario: 54 yo alcoholic homeless woman comes in with complaints of diffuse abd pain assoc with N/V and HA which started after having had one pint of vodka the night before. She has been seen for the same exact scenario in the ED 2 days prior. At that time, NL labs and abd xrays. Pt treated with dilaudid and phenergan. Feels better, the goes home. On this presentation, she immediately starts asking for pain meds to help her feel better. Looking back at her chart, you notice she’s been worked up 3 times this year for the same exact symptoms all of which started with her drinking. Each time, her work up was normal and she improved with dilaudid/phenergan. It was assumed she either had alcohol related gastritis or she was just drug seeking.

Ethical question: I know it’s our duty to do no harm and to treat everyone equally. Thus, we’re obligated to work this woman up like it’s her first visit and treat accordingly, but I can’t help but get irritated with the situation. People, who seemingly abuse the system by continuing to inflict damage on themselves knowing someone else will pay for their irresponsibility. I know inherently this statement is judgmental as I’m sure there are a complex web of reasons that have led her to this point, none of which I can relate to or begin to judge. Nevertheless, I think we see a lot of these scenarios where people abuse the ER because they’re not held accountable for their actions. I’m not talking about people that don’t have healthcare and thus no access to the system, but rather people who continue to ignore medical advice or inflict damage on themselves, and yet return to the ED for medical care. Thoughts?

But Do You Like Him?: I have had really good interactions with all of the attendings that I have had this month. One of them (that I really enjoy working with) had an interesting way of triaging the severity of a patient's presentation. I would see the patient, gather a history and perform a physical. Then, I would present the patient to the attending. Often, the complaint would be something that was probably minor but could be more serious - abdominal pain, nonspecific chest pain, fall without bruising, etc. His first question for three of these such patients in a row was "So, do you like him/her?" As if to say that my reaction to the patient and my degree of sympathy/empathy for them determined how much workup we would perform.  Again, I do really like this doc and feel that he did give everyone an appropriate workup in the end, I just found it interesting that this was a key part of my presentation.

ER as a Waiting Room: Recently I had a patient that was sent to ther ER by a specialty physician's office in order to be seen by that physician.  When I first saw her she was very resistant to giving the H&P because she was there to "just see my doctor".  Upon further investigation, it became clear the specialist had never seen her before. A resident was eventually dispatched (after I called that team's pager to see when he thought he would be seeing her because she was thinking of walking out and was berated).  this seems like an incredibly inefficient use of the ED since what this patient needed was an outpatient appointment; additionally, she did not want to be evaluated by any of the ED physicians.  This whole situation was very awkward because we wanted to do right by the patient but using the ED as  a waiting room for specialist appointments is incredibly wasteful.

Ohio Medicaid / Caresource Patients: Recently, Ohio Medicaid informed its insured that many of the Columbus hospitals would no longer be covered under their benefit plan (such as the XXX Health Systems).  I believe XXX, XXX and XXX's are among few that patients can be sure that the cost of being seen would be covered!  While this does prevent every patient in need of services from going to any hospital or the closest emergency room for treatment, it adds a great deal of financial worry/stress to both the provider and the patient guarantor.  I was covering a shift this week and one of the FLAGS in the system included the patient insurances.  In this case, a patient had Medicaid and was about to be admitted.  There was a delay in full admission and subsequent evaluation as several people were unclear as to "which service the patient should be admitted to with Medicaid."  At some point the House Supervisor and Nurse Manager was contacted and provided the information and the patient was admitted.  Most times the hospital just transfers the patient to XXX (I've seen this happen!)This is a matter that we as professionals do not have much control over.  We are influenced by changes in the healthcare system so much that it changes the flow of our day-to-day practice.  Are we delaying care or helping the patient avoid medical debts? Providing less effective care to Medicaid holders?  How does this patient flow toward the covered hospitals impact XXX’s doctors, students, patients and other insurance holders that seek beds at XXX?

Less is More: Today I had a 43 yo female pt that c/o acute chest pain that awoke her from sleep. She had all the typical symptoms that would warrant a workup for acute MI. Her EKG was normal and her cardiac troponins were negative.  She continued to c/o increased chest pain and dypsnea so we ordered a d-dimer which was slightly elevated. She then got upgraded to the CDU and got a CT-angio that was normal.  Throughout this entire workup she continued to ask for more pain medication as she was getting increasingly anxious. During my examination her husband would ask specific medical questions regarding the tests we were ordering, such as "what is a cardiac troponin?", "what is a STEMI?", "why are you ordering a d-dimer, do you think she has a PE?". In the meantime this made the pt exceedingly anxious as I tried to explain these medical terms and tests in laymans terms. I could sense there was friction between the couple and that his presence sparked her anxiety.  I also noticed an unusual co-dependency that existed. It seemed the husband was pre-occupied with her care, almost as if he got a strange satisfaction from being "fatherly figure" in her life.  On the other hand, she had a complex PMHx of fibromyalgia, anxiety, depression, and RA and seemed to depend on his "fatherly care" as the one constant in her life. As more and more tests became normal and we were more confident in her stable status, we amazingly convinced the husband to take a lunch break to see if the pts anxiety improved. Sure enough, with her husbands absence the pts chest pain rapidly improved. In retrospect, I think had I identified this strange anxiety provoking co-dependence that existed here I would have been more akin to provide less medical explanations and wait until the test results made the diagnosis more clear. This would have prevented the husband from inflicting more anxiety and worsening chest pain in his wife.  I learned that sometimes, less information is more.

**Professionalism Narrative: Teaching-and-Learning Domain**

**Major Theme: Creating an (un)welcoming environment**

**Sub-category: Respecting colleagues/learners from lower hierarchies**

Professionalism in a stressful situation: I don't know how most of your code/serious medical situation have gone in medical school so far, but for me, usually there are way to many people in the room and eventually someone yells at me (and others) to get out of the room because your just standing there watching.  And while I understand that they need room, I would like to stay there and process the whole situation, so that when I have to run one of these, I feel somewhat prepared. Well on my last shift, we had a patient who presented with stroke like symptoms.  The patient was getting set up in the room (nurse assessment, monitor, IV, etc.), but she was starting to decompensate.  The attending and I went to the room, and it was apparent that we need to intubate the patient.  So here we were in this tiny room with 2 nurses trying to start IVs, one nurse that was charting, the attending, respiratory therapy, pharmacist, a tech to do an EKG, and me.  But this time instead of getting kick out of the room, everybody took the time to explain things to me.  The attending went over rapid sequence intubation as he was performing it, and afterwords we talked about the different instruments in the intubation "tackle" box.  The pharmacist also called me over afterwords and explained all the medications and indications in the intubation box. Overall, it was a very good learning experience for me, and I wanted to thank everyone for letting me be apart of it instead of being designated to the halfway.

Yelling: last week I was doing a 11-7 and just before the end of my shift we got a call for a trauma I. My resident and I responded to the call and waited for the patient to arrive. The patient arrived and we began the primary assessment. It was at this time that the supervising attending arrived on the scene. Now I must put a disclaimer that I don't know what kind of day this doctor was having but he started yelling at the residents, "your going to slow", you should have had that done 5 minutes ago" creating allot of stress in an already stressful situation. Needless to say. the code ran poorly and I observed that it ran even worse after the yelling began. After the code my resident and I talked about the situation and he told me that was inappropriate, I agreed. Yelling is unprofessional in any situation. If yelling can get people fired in other professions then why do so many people in the health field feel that they have permission to do so? As I observed that day yelling gets you nowhere, lesson learned.

Attending Attitudes: This discussion could get dicey if managed incorrectly - something I recently was told medical students do quite often in the E.R. - so please proceed with caution. We all have worked on varied teams composed of different personalities and know the attitudes of our coworkers are inherently vital to the success of the group as well as to our own learning. Over the last two years I have worked with enough (albeit, thankfully, *only* *a few*) unpalatable attending physicians to know my own ever-predictable response: become frustrated, mouth off, cease taking initiative, stop reading, receive poor feedback, regret mouthing off, lather, rinse, repeat. With the full knowledge my own attitude toward these unsavory individuals is in need of drastic refinement, I must comment on how pleasurable it is to work with attendings and residents who truly enjoy teaching and who take a few extra seconds to inquire about me, my interests, and my residency plans. Granted, these are the "easy" situations, and one likely would have to be socially challenged not to appreciate that particular scenario, But the creation of such an experience reflects greatly on the personal and professional development of the supervising physician - the ability to direct and lead a group of individuals through the highly complex process of achieving flawless patient care while still maintaining a humble, serving attitude is (to me) the consummation of a successful integration of knowledge and compassion. That is my ideal, and I truly appreciate the efforts of those who aspire to this end.  Feel free to comment. Of course, if you disagree, expect a good mouthing off in return. :)

Lack of Professionalism: Throughout my shifts in the ED, I have worked with many different attendings, all who have been great teachers and very receptive to me as a medical student. However during my last shift, I had overheard another attending speaking to the attending that I had been working with for that day.  First, he was badmouthing the residents who had been working that shift and then he said that medical students were completely worthless and he couldn't stand working with them.  This was said in a loud voice in front of me, the resident that I was working with and rooms of patients.  Even after my attending pointed out that I, a medical student, was standing right there, he merely looked at me and stated that he "wasn't going to apologize, that's the way it is."   Now, I agree at times we, as medical students, cannot contribute as much as we would like.  We can't put in orders, we can't do procedures on our own, and sometimes we even ask questions or would like to be taught.  However, we will never become useful, competent physicians without going through this learning experience.  If this particular physician thinks that it is not worth his time to work with medical students, then maybe he should not be at an academic institution or he could at least not let his opinion be know so loudly if front of patients who are being cared for by residents and medical students.  Like I said before, every physician that I have personally worked with in the ED has been very professional and welcoming to medical students and I hope that this is just an example of one bad seed.

You Never Know Who is Listening: There's nothing too much more bothersome to a medical student than when their superiors seem to not care to deal with them.  While in the ER, I overheard a doctor ask another doctor about whether they were warming up to staffing patients.  The doctor responded, "yeah, it's ok, but I don't really like to staff with med students."

Pimping in Front of Patients: Pimping....it's ubiquitous in medical education. Regardless of your taste or distaste for the ritual, it has survived for generations, and it likely won't go away any time soon. The (benevolent) goal of pimping is education. As we are in a field of "lifelong learning", we should all be open to the offer of further education...even if it comes in the form of pimping. But I think there are times when pimping is inappropriate and/or done with intentions other than to increase knowledge. As medical students in the clinics, we have the difficult job of balancing competence in service to our patients and continuing our education. And I think that pimping in front of our patients CAN undermine the small amount of credibility (and perhaps trust/rapport/etc) that we have established in our initial solo encounter with that patient.

Certainly this is a case-by-case basis comment. But having experienced this for the past two years, including on this rotation, it merits some thought from each of us as we progress into the next stage of our training......where WE may be the ones pimping.My thoughts?1) Ensure that there is a valid and IMMEDIATELY PERTINENT educational point to be made, when pimping in front of the patient. Otherwise, you could a) phrase it as a statement rather than a question or b) ask the student when you've left the room.2) Ensure that the WAY you phrase the question is not demeaning or clearly an attempt to stump the student --- this especially undermines the competence/credibility of the student in their attempt to provide a meaningful contribution to the care of this patient.3) Don't forget about the patient during your conversation --- this makes the patient feel like a learning tool, and further defines the student as incompetent and inexperienced (in the eyes of the patient, this may lead them to believe that the student serves no role in their care). Try to include the patient or patient's experiences in the conversation, further eliciting symptoms or relating to their personal story......makes the pimping seem less punitive AND it helps educate the patient too. Now certainly, I have only witnessed a couple of egregious examples of pimping in front of the patients, but I think we should aim to not just avoid "egregious violations", rather we should try to envision the "ideal" interaction when attempting to help educate via pimping and strive for that.

**Sub-category: Included and acknowledged as a medical student**

Attitudes Can Make All The Difference in Medical Education: I struggled with mixed feelings about my experience in the ED this month. I worked with some attendings that were my absolute favorites in my whole medical school experience. I worked with others that were not my favorites at all. I've wondered a lot about that. I know that the ED is a busy place that can be particularly difficult to involve a medical student in, but I also know that it can be one of the most educational places in the hospital. Sometimes I felt like I was involved in every teaching opportunity that was present in the ED throughout the entire shift. Other times I felt like I was a little more than an annoyance to be tolerated. I tried to assess my own attitude to see if that was what was making the difference. I looked for a pattern with certain attendings to see if it might be their attitude that was making the difference. I also looked for patterns in how busy the ED was on any particular day. My conclusion was that the difference between a great shift and a not-so-great shift was unrelated to how busy the ED was. I truly feel that the difference was due to a combination of the attending's attitude and my attitude. I'm sure there were shifts where I wasn't as aggressive or proactive as I could have been. But it was truly difficult when I felt like attendings would avoid my glance for fear that I would want to present a patient to them. As such, I'm also sure that there were shifts where the attending I was with could have been more excited about having a student along.This latter fact was firmly established in my mind by a certain attending in the ED at XXX who always made each of my shifts amazing when I was with him. His enthusiasm for patient care and education was contagious. His desire and ability to teach is remarkable. I do count myself fortunate to have worked with him, and hope that I remember to be more like him when all is said and done rather than like those whose attitude contributed to some long shifts for me. In the end, the only thing that any of us have control over is our own attitude. Some day we'll be attendings. Then it will be our attitudes that can make such a significant difference in the day of some medical student. Let's not forget it.

**Major Theme: Capitalizing on teaching opportunities**

**Sub-category: A leader who teaches-asks questions, explains, spends time, learns**

Facebook in the ED: The other day in the XXX's ED, one of my attendings began checking her facebook page during a slow morning. She probably stayed on facebook for about 45 minutes, talking about it with other staff members and looking at other people's pages. While this activity was not taking away from that person's clinical responsibilities (since there were no new patients), I found it unprofessional for several reasons. First, it sets a bad example for residents and medical students about proper internet use in the workplace. Second, in an open computer bay in the ED, patients and families can easily see your computer screen. If I were a parent, I might find it disturbing to see my child's doctor checking their facebook page during the ED shift. Conversely, if I were a child, I might even try to search that person on facebook, which may result in access of personal information, pictures, etc that may compromise the patient-provider relationship. Apart from the professionalism issue (and this is just me being obnoxious), I don't think this was great use of down time in the ED. I always appreciate those attendings who take advantage of down time to teach students and residents or to address questions about patient care decisions. I like facebook just as much as the next person, but I've observed this scenario multiple times at various hospitals. I'm curious whether anyone has a different opinion or experience with this.

**Sub-category: Using opportunities to teach values and manners**

Dealing With Belligerent Patient: One of the most difficult times to deal with a patient is when they start becoming belligerent and acting in a violent or disrespectful manner.  It is very easy during that time to get angry with the patient and start to act in a similar manner back toward them.  One day in the ED recently my attending and I had a patient that had been drunk and had cut himself.  He had to be placed into restraints because before arriving for my shift he had swung at some of the staff in the ED.  He started sobering up by the time we arrived and was fairly pleasant while we were doing our part to sew up his arm.  Shortly thereafter the patient once again became angry and somehow got out of his restraints.  Multiple staff were holding him down while he was lashing out and using very vulgar language.  At that time my attending took a moment to go over to the patient and try to calm the situation.  He began to explain to the patient that what he was doing was going to continue to keep him restrained and he was not helping himself.  He did a good job at calming the patient and we then had an opportunity to talk about the situation.  He admitted that when he was first coming out of residency he would get hot headed and start lashing back at patients, but he has since realized that it reflects poorly on the department and in the end gets you no where with the patient.  It is better to remain calm and remember that there are medications on your side that can help diffuse the situation.  It is easy to lose your cool in these situations, but it is always better to take a step back, relax, and do your best to remain as professional as possible, no matter how the patient acts.

**Sub-category: Giving safe and structured responsibilities**

Accruing procedure skills as a medical student. This post concerns neither a positive nor lapse in professionalism, but is a discussion on how we as med students represent our qualifications to patients (or residents) prior to performing invasive procedures. There were numerous examples over the past month, but I will name just two of them. (1) Neurosurgery resident was tending to a sick patient who had a SAH, that required urgent ventriculostomy at the ED. He mentioned a need to place an art line. Being an eager and confident Med-4, I immediately offered my services. The n/s resident turned to me, and asked how many such lines I had successfully placed in the past, to which I replied "One". He politely declined, citing that he did not feel comfortable. (2) A patient presented with two emergent scrotal abscesses that required I&D. I followed him throughout his ED stay, making sure he got his Dilaudid, and informed him the exact nature of the procedure. But when the time came to numb and incise, he told me that he wanted the resident to do it instead due to the delicate parts down below. The ED resident agreed to perform the I&D herself, which was obviously the right thing to do (patient autonomy and quality of care). The patient then asked the resident whether she had done many of these (i.e. scrotal abscesses), to which she replied "I do this all the time". There are two competing forces at work here. One is the need to practice procedures in order get good; the other is presenting one's qualifications accurately. Accruing procedures is a classic "Chicken and the Egg" conundrum. You can't get good at something if you don't practice, but patients (or residents) may not want you to do a procedure if you haven't done it before. Nonetheless, I think honesty is the best policy. If anybody asks me point-blank how many times I've done such-and-such a procedure, I will say the actual number. However, if the questioning is more vague, I'll probably pull a Clinton - "Don't ask, don't tell." Then, of course, there is always a temptation to fib (ie inflate the figure) and get the job. In the first case, I considered the possibility the n/s resident might have let me place the art line if I fibbed and told him I'd already placed over 10 art lines (which might be true two months from now, after my ICU rotation at Riverside). The second case was more straightforward in that the patient wanted the resident to do it. But there is an interesting hypothetical here: what if as a resident next year, let's say with 50 I&Ds under my belt, but just 1-2 cases of scrotals, a similar patient specifically asks me how many scrotal abscesses I've done. How would I respond? The choices are: - tell the truth (i've done 2 so far in my career) - fib (I've done too many to count)  - redirect (oh, don't worry about it, this one's in the bag. I know what I'm doing).I'm curious what some of you think, either about these particular cases or in general about accruing procedure skills.

Teaching Hospital: One aspect of being a medical student that I find difficult is being able to learn new procedures.  This is a tough thing because our future patients are going to want us to be proficient in what we are doing.  The only way to achieve this is through practice.  But at the same time no one wants someone to be practicing on them.  Recently while performing a LP and the patients mom asked if I had done many of these.  I said that I have practiced them many times on cadavers or models.  As I proceeded through the procedure the mom and patient became more and more agitated that I was able to attempt the procedure.  I felt somewhat awkward because I was not proficient in the procedure yet I was attempting it.  But at the same time when will I learn if I don't learn now.
